# The impact of biologically relevant negative samples on machine learning-based B-cell epitope prediction for Influenza A

**DOI:** 10.1093/bioadv/vbag127

**Published:** 2026-05-04

**Authors:** Claudia Pareja-Barrueto, Jose Reyes-Suarez, Felipe Del Canto, Felipe Besoain, Renzo Angles, Mauricio Arenas-Salinas

**Affiliations:** Department of Haematology and Oncology, Pontificia Universidad Católica de Chile, Santiago, 8330077, Chile; Centro de Bioinformática, Simulación y Modelado (CBSM). Facultad de Ingeniería, Universidad de Talca, Talca, 3460000, Chile; Núcleo Interdisciplinario de Microbiología, Instituto de Ciencias Biomédicas, Facultad de Medicina, Universidad de Chile, Santiago, 8380453, Chile; Department of Interactive Visualization and Virtual Reality, Faculty of Engineering, Universidad de Talca, Talca, 3460000, Chile; Department of Computer Science, Faculty of Engineering, Universidad de Talca, Curico, 3344158, Chile; Centro de Bioinformática, Simulación y Modelado (CBSM). Facultad de Ingeniería, Universidad de Talca, Talca, 3460000, Chile

## Abstract

**Motivation:**

Predicting linear B-cell epitopes remains a major challenge in immunoinformatics, particularly for rapidly evolving viruses such as Influenza A. Many existing predictors rely on heterogeneous training datasets, poorly defined negative samples, or low-interpretability models, which can limit performance on pathogen-specific tasks. Improving prediction therefore requires biologically meaningful datasets together with informative and interpretable sequence representations.

**Results:**

We developed a machine learning framework based on sequence-derived physicochemical descriptors for linear B-cell epitope prediction in Influenza A. A curated dataset of experimentally validated epitopes and non-epitopes was constructed using redundancy reduction and balanced sampling strategies. Five supervised classifiers were evaluated, and the effect of real versus artificial negative datasets was systematically assessed. Feature selection using Analysis of Variance and Mutual Information showed that predictive performance emerged from the combined contribution of multiple descriptors rather than single variables. The best model, Random Forest, achieved 82% accuracy, 83% F1-score, a Matthews correlation coefficient of 0.65, and an area under the receiver operating characteristic curve of 0.90. Benchmarking against widely used tools showed improved balanced performance on our curated Influenza A dataset.

**Availability and implementation:**

Source code, processed datasets, and reproducible analysis scripts are freely available at GitHub: https://github.com/cparejabarrueto/epitopes.

## 1 Introduction

The adaptive immune system relies on the precise recognition of antigenic determinants, or epitopes, by B and T lymphocytes. In particular, B-cell epitopes (BCEs)—regions of antigens recognized by membrane-bound immunoglobulins (BCRs) on B cells—are essential for initiating humoral immune responses.

**Figure 1:**
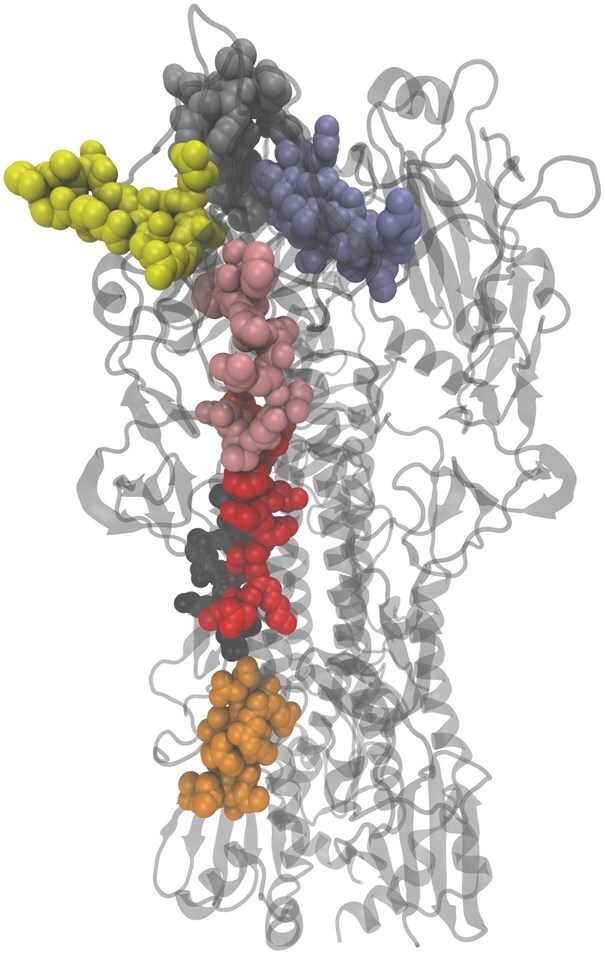
Structure of the hemagglutinin from influenza virus A (PDB ID: 1MQL). In sphere are represented some epitopes used in this study.

BCEs are generally divided into two main types: continuous (or linear) and discontinuous (or conformational). The continuous BCEs consist of sequential amino acid residues of an antigen. In contrast, discontinuous BCEs are composed of amino acids that are not sequential in the protein sequence but are proximal in the 3D structure of the folded protein (Manavalan *et al.* 2018), as illustrated in Fig. 1. Among them, linear BCEs, which consist of continuous stretches of amino acids, are widely used in synthetic peptide vaccines, diagnostic assays, and antibody production strategies.

Accurately identifying these sites on antigenic proteins is a crucial step for vaccine development, in order to design better immunogenic molecules for targeted therapies, to predict cross-reactivity, and/or to gain insights into the antigen–antibody interactions (Terajima *et al.* 2013, Mendes *et al.* 2022, Yurina and Adianingsih 2022, Zhou *et al.* 2025). With the revealing threat of emerging and re-emerging infectious diseases, swift and effective epitope mapping has become indispensable to devise effective therapeutic and prophylactic responses (Purcell *et al.* 2007, Cai *et al.* 2021, Zeng *et al.* 2023).

One of the viruses of greatest interest for vaccine development is the influenza A virus, which belongs to the Orthomyxoviridae family. It is a principal culprit behind flu epidemics and has historically orchestrated numerous pandemics, including the devastating 1918 Spanish flu (Taubenberger and Morens 2006, Short *et al.* 2018). The virus’s hallmark is its ability to infect numerous species, including humans, birds, pigs, and horses, among others, and its rapid adaptation, facilitated by two phenomena: “antigenic drift” and “antigenic shift,” poses a continual public health challenge (Webster *et al.* 1992). It is estimated to be responsible for between 300 000–650 000 deaths worldwide each year (Kyu *et al.* 2018).

Influenza A strains are categorized based on their surface proteins, hemagglutinin and neuraminidase. While 18 different H subtypes and 11 N subtypes have been identified in aquatic birds, only certain combinations like H1N1 and H3N2 have been established in the human population (Tong *et al.* 2013, Paules and Subbarao 2017, Javanian *et al.* 2021). Annual vaccination remains the leading preventive strategy against influenza, though the continually changing nature of the virus necessitates periodic review and updating of vaccines (Osterholm *et al.* 2012, Nypaver *et al.* 2021, Kumari *et al.* 2023).

Influenza A virus contains several antigenic proteins that elicit immune responses, primarily through interactions with host antibodies. The most immunodominant surface proteins are hemagglutinin (HA) and neuraminidase (NA), which facilitate viral entry and release, respectively, and are the main targets of neutralizing antibodies and seasonal vaccines. In addition, internal and structural proteins such as the matrix protein 1 (M1) and membrane protein 2 (M2) have also been shown to contain BCEs. While less studied, non-structural proteins like NS1 and NS2 have also been implicated in modulating host immunity and may present immunogenic regions. Understanding and characterizing epitopes within these proteins is essential for guiding epitope-based vaccine design and enhancing the effectiveness of immunoinformatic models.

Precise identification of epitopes is challenged by the inherent complexity of molecular interactions and their associated structural and functional context (Li *et al.* 2020). While experimental methods, like X-ray crystallography and nuclear magnetic resonance, have been primary tools for epitope identification (Davies and Cohen 1996), they can be costly, slow, and technically challenging.

The increasing interest in developing and optimizing computational methods to predict epitopes is stimulated by the abundance of available protein sequence data and advances in machine learning (Sun *et al.* 2013, De Groot *et al.* 2020, Raoufi *et al.* 2020, Yurina and Adianingsih 2022). Bioinformatic-based prediction algorithms promise epitope identification at a pace and cost significantly lower than traditional approaches. These methods, ranging from algorithms based on physicochemical properties to more sophisticated techniques employing neural networks and deep learning, have proven promising in accurately identifying epitopes across diverse systems (Dhanda *et al.* 2013, Jadid and Habibi 2017, Solihah *et al.* 2017, Xie *et al.* 2018, Kuo *et al.* 2020). Immunoinformatics, emerging as a crucial interdisciplinary field, has proven to be a valuable tool in vaccine design (Ali *et al.* 2017, Z. Yang *et al.* 2021, Bravi 2024, Wei *et al.* 2024), epitope identification, understanding antigen–antibody interactions (Abbott *et al.* 2014, Hu *et al.* 2014, X. Yang *et al.* 2021), and analyzing the diversity of the lymphocyte repertoire (Brusic and Petrovsky 2005, Dangi *et al.* 2016).

Although various computational tools for BCE prediction have been developed, many of them rely on general-purpose models trained on highly heterogeneous datasets that include epitopes from multiple pathogens. This lack of specificity often limits their predictive performance when applied to fast-evolving viruses such as Influenza A. For instance, approaches like Epitope1D demonstrate high accuracy across diverse taxa by using only a small set of interpretable descriptors (da Silva *et al.* 2023). Recent advances in epitope prediction have increasingly incorporated protein language models (PLMs) such as ESM, ProtBert, and TAPE, which learn rich sequence embeddings capturing contextual and evolutionary features. These embeddings have shown strong performance in BCE prediction tasks, often when combined with neural architectures or hybrid feature selection frameworks (e.g. MLAFP-XN, Epitope1D, deepBCE-Parasite, EpitopeVec (Bahai *et al.* 2021). However, such models typically require extensive computational resources and are often considered “black box” systems, limiting interpretability. In contrast, our study focuses on interpretable, sequence-derived molecular descriptors that provide direct physicochemical insight into the determinants of epitope recognition, while still achieving competitive predictive performance.

In addition, the definition and selection of reliable negative datasets remains an unresolved challenge in epitope prediction studies, potentially introducing bias during training. To address these gaps, we present a virus-specific approach that integrates interpretable molecular descriptors and machine learning classifiers to predict linear BCEs from amino acid sequences of Influenza A proteins, using both real and artificial negative datasets. This strategy aims to improve prediction accuracy and offer a robust framework for epitope-driven vaccine design.

Our research focuses on exploring machine learning and AI methodologies evaluating the impact of the selection the dataset (positive and negative) and molecular descriptors on the performance of the models, comparing with other existing software. A key innovation of this study lies in assessing how the composition and origin of negative data influence the predictive behavior and overall performance of the models. By systematically comparing these datasets, we aimed to highlight their critical role in optimizing the identification of novel BCEs and to address a largely underexplored variable in epitope prediction workflows.

We also included non-structural proteins in our analysis to broaden the search space for potential antigenic regions. Although structural proteins are traditionally associated with antibody responses, recent studies have shown that non-structural viral proteins can elicit B-cell responses, especially in the context of viral replication and infection dynamics. Therefore, their inclusion allows the model to learn from a more diverse antigenic landscape and potentially identify epitope candidates. This approach aligns with current trends in immunology that seek to explore less-characterized targets for vaccine and diagnostic development. We focused exclusively on linear BCEs, as these can be predicted directly from amino acid sequences without requiring structural data. Linear epitopes are widely used in peptide-based vaccine design and immunodiagnostics due to their accessibility and synthetic feasibility.

## 2 Methods

### 2.1 Data source and preprocessing

#### 2.1.1 Source and selection of epitopes

All epitope sequences were obtained from the Immune Epitope Database (IEDB) (http://www.iedb.org) (Vita *et al.* 2019), selecting experimentally validated linear BCEs from Influenza A proteins, using specific criteria: linear epitopes as molecular structure, influenza A virus as the source of the epitope, T and B lymphocytes, MHC ligand in assays, humans as hosts, and infectious diseases. The proteins used included Polymerase (UNIPROT IDs: P03428, P03431, P03433) (Fields and Winter 1982), Hemagglutinin (P03436) (Both and Sleigh 1980), Nucleoprotein (P03466) (Winter and Fields 1981), Neuraminidase (P03468) (Fields *et al.* 1981), Matrix protein (P03485, P06821) (Winter and Fields 1980), Non-structural protein (P03496) (Baez *et al.* 1980), Nuclear export protein (P03508) (Baez *et al.* 1980), and Protein PB1-F2 (P0C0U1) (Winter and Fields 1982).

From these sources, a total of 256 amino acid sequences were compiled for the **positive dataset**. The peptides ranged in length from 8 to 52 amino acids. All epitopes were confirmed to be unique following a redundancy check.

#### 2.1.2 Redundancy control

To assess the influence of sequence redundancy on the performance of epitope classification models, we applied the CD-HIT clustering algorithm to reduce similarity within the datasets. Redundancy filtering was conducted independently on both the positive (epitope) and negative (non-epitope) datasets, using the complete pool of available samples for each prior to any partitioning. We employed identity thresholds of 95%, 90%, 85%, and 80%. For each threshold, sequences sharing more than the specified percentage identity within their respective class were clustered, and a single representative was retained per cluster. The number of remaining sequences at each threshold is summarized in Annex 7, available as supplementary data at *Bioinformatics Advances* online.

This intra-class filtering step was intentionally performed on the total data before the 80/20 split to guarantee that the training and test sets remained strictly non-redundant relative to each other. This sequence of operations was chosen to prevent the inclusion of highly similar sequences in both partitions, a condition that would otherwise result in an over-optimistic evaluation of the model’s generalization capabilities. By establishing this non-redundant baseline at the outset, we ensure that the test set serves as a truly independent and challenging benchmark for performance evaluation.

Following redundancy reduction, the final non-redundant dataset was divided into training and independent test sets using a stratified 80/20 partition. All subsequent model development procedures, including feature selection and hyperparameter tuning via cross-validation, were carried out exclusively within the training subset. This ensures that the test set remained completely unseen during the model optimization and selection processes, maintaining the integrity of the final evaluation.

#### 2.1.3 Construction of Negative datasets

In this study, we utilized two distinct negative datasets.

##### 2.1.3.1 Negative Set A (real)

Protein regions from Influenza A that were experimentally tested and confirmed not to elicit antibody binding (non-epitopes) in IEDB, identical to the one used by Singh *et al.* (2013), consisted of unique non BCEs experimentally validated, amounting to a total of 1795 samples.

##### 2.1.3.2 Negative set B (artificial)

Was constructed using the protein sequence of virus influenza, but the described epitopes sequence by IEDB database was removed. To standardize peptide length for feature extraction, all sequences were segmented into overlapping 14-residue windows. Sequence segments not identified as epitopes were divided resulting in a total of 4312 fragments. Epitopes are typically considered to be between 11 and 17 amino acids in length (Paul 2012) and many epitope prediction approaches employ peptide windows in the range of ∼12–16 residues to capture the minimal binding region together with flanking residues that contribute to antibody recognition (Jespersen *et al.* 2017). A window size of 14 residues therefore represents a biologically reasonable compromise that preserves relevant sequence context while maintaining a consistent representation for machine learning feature extraction.

This dual approach allowed us to assess whether biologically meaningful negatives improve model discrimination compared with synthetic sequences, addressing a limitation noted in previous epitope prediction studies.

#### 2.1.4 Stratified dataset splitting

The clustered dataset was divided into an 80% training set and a 20% independent test set using a stratified splitting strategy, ensuring that the original 1:1 ratio of positive to negative peptides was preserved in both partitions. This approach maintained consistent class proportions across subsets and supported reliable performance evaluation. Importantly, the test set was not used at any stage of model selection, feature engineering, or hyperparameter optimization.

For each dataset (positive, negative A, negative B), we calculated several molecular descriptors. Since the epitope fragments vary in length, we only utilized normalized descriptors. Finally, we obtained a total of 62 attributes for each amino acid sequence, including positive and negative datasets.

Each piece of data underwent rigorous preprocessing and cleaning to ensure accuracy and reliability in our findings. The final clean dataset, enriched with valuable insights and without irregularities, is readily accessible via the repository link provided in GitHub.

### 2.2 Molecular descriptors

The epitope fragments obtained were analyzed using molecular descriptors derived from their amino acid sequences. These descriptors were computed with the Peptide package available in R, as described by Meher *et al.* (2017) and Osorio *et al*. (2015).

Descriptors encompassed:

- Physicochemical properties (hydrophobicity, charge, polarity, flexibility, isoelectric point, molecular weight, instability indices, hydrophilicity).
- Structural tendencies (secondary structure propensity, surface accessibility).
- Compositional descriptors (count of amino acid, specific compositions and dipeptide frequencies).

A total of 62 attributes were used. Annex 6, available as supplementary data at *Bioinformatics Advances* online shows the explanation of these selected descriptors.

#### 2.2.1 Selecting relevant molecular descriptors and cumulative attribute evaluation

Sequence-derived descriptors were analyzed and ranked using Mutual Information and ANOVA-based feature selection methods. The resulting ranked lists were evaluated through a cumulative performance approach, following the methodology described in Muñoz *et al.* (2024).

### 2.3 Feature selection

Two feature selection approaches were implemented to reduce dimensionality:

- **ANOVA F-score**: Used to identify descriptors with the strongest discriminatory power between classes.
- **Mutual Information (MI)**: Assessed the contribution of each descriptor to the prediction of immunogenic epitopes by measuring the dependency between variables

The **top 20 most relevant descriptors** were selected based on cumulative predictive performance (Annexes 3 and 4, available as supplementary data at *Bioinformatics Advances* online). The final set of descriptors was obtained by merging the unique descriptors from both selection approaches.

### 2.4 Machine learning model construction and evaluation

#### 2.4.1 Implemented algorithms

Five supervised classification models were evaluated, Support Vector Machine [SVM], Naive Bayes [NB], Random Forest [RF], XGBoost [XG] and Decision Trees [DT]) for the prediction of BCEs for Influenza A viruses, as described in Muñoz *et al.* (2024).

The configuration of the software used is available in Annex 5, available as supplementary data at *Bioinformatics Advances* online.

#### 2.4.2 Hyperparameter tuning

Hyperparameter optimization was performed using grid search within the five-fold cross-validation procedure applied exclusively to the training subset. Predefined parameter grids were explored for each algorithm following commonly used practices in machine learning applications to biological sequence analysis. The optimal hyperparameter configuration for each model was selected based on the average cross-validation performance. The complete parameter grids evaluated and the final configurations used for model training are provided in Annex 16, available as supplementary data at *Bioinformatics Advances* online to ensure reproducibility of the analysis.

All analyses were conducted in Python 3.10 using scikit-learn (v1.4) and XGBoost (v2.1). Descriptor generation and feature selection were performed in R 4.3. The computational pipeline was executed on an Ubuntu 22.04 system equipped with an Intel i9 processor and 64 GB of RAM.

#### 2.4.3 Training, balance and cross-validation procedure

To assess the generalization ability of the models while preventing data leakage, the dataset was partitioned into 80% training and 20% independent testing subsets using a stratified split that preserved the original class proportions. This train–test partitioning was repeated five times using different random seeds to evaluate the stability of the modeling pipeline. In all cases, the independent test set was reserved exclusively for final model evaluation and was not used during model training, cross-validation, or hyperparameter optimization.

Model development was conducted solely on the 80% training subset. Within this subset, a five-fold cross-validation strategy was applied for model training and validation. During this process, hyperparameter optimization and feature-related decisions were performed exclusively using the training data.

To address potential class imbalance during model training, a data balancing strategy was applied within the training subset. Specifically, random undersampling of the majority class was performed to construct balanced training sets. Due to the stochastic nature of this procedure, the undersampling process was repeated 100 times using different random seeds to assess the stability of the training process. Across these repetitions, model performance and selected hyperparameters showed minimal variability, indicating consistent and robust behavior.

Following cross-validation and parameter selection, the final models were retrained using the complete 80% training data and subsequently evaluated on the independent 20% test set. This procedure ensured that all cross-validation results reflected internal model consistency, while the reported performance metrics correspond exclusively to the independent test evaluation.

#### 2.4.4 Prevention of information leakage

To ensure strict separation between training and evaluation data, all redundancy reduction (CD-HIT clustering), feature scaling, feature selection, and hyperparameter optimization steps were performed exclusively within the training subset or its cross-validation folds. The independent test set remained untouched until final model evaluation. This workflow was designed to prevent information leakage arising from sequence similarity, preprocessing transformations, or model selection procedures.

### 2.5 Model performance evaluation

#### 2.5.1 Performance metrics

Model performance was assessed using standard classification metrics, including accuracy, precision, recall, F1-score, Matthews correlation coefficient (MCC), receiver operating characteristic area under the curve (ROC-AUC), and precision–recall area under the curve (PR-AUC). Accuracy reflects overall classification correctness, while precision and recall quantify the ability to correctly identify positive instances and avoid false positives, respectively. The F1-score summarizes the trade-off between precision and recall, and MCC provides a balanced measure of binary classification quality that accounts for all elements of the confusion matrix. ROC-AUC and PR-AUC were used to evaluate the discriminative performance of the models across decision thresholds.

Performance metrics were computed on the independent test sets and summarized across repeated train–test splits to assess the robustness and stability of model performance. Formal definitions of the confusion-matrix–based metrics are provided below.


(1) $$\mathrm{Accuracy}=\frac{TP+TN}{TP+TN+FP+FN}$$

(2) $$\mathrm{Recall}=\frac{TP}{TP+FN}$$

(3) $$\mathrm{Precision}=\frac{TP}{TP+FP}$$

(4) $${\;F}_{1}=\frac{2\cdot \text{Precision}\cdot \text{Recall}}{\text{Precision}+\text{Recall}}$$

(5) $$\;\mathrm{MCC}=\frac{\left(TP\cdot TN)-(FP\cdot FN\right)}{\sqrt{\left(TP+FP)(TP+FN)(TN+FP)(TN+FN\right)}}$$

### 2.6 External robustness tests

To evaluate the generalization capability of the best-performing model (RF), we conducted additional tests on three independent datasets:

- Mutated epitopes: we assessed the model’s sensitivity to sequence variation by testing linear BCEs from Influenza A that were progressively mutated at 10%, 20%, 30%, 50%, and 70%. This allowed us to quantify how well the model recognizes immunogenic patterns under increasing divergence.
- Reversed epitopes: inverted sequences to test sequence-order sensitivity.
- Cross-viral dataset: experimentally validated BCEs derived from the Dengue virus to assess inter-viral transferability.

These complementary evaluations enabled us to characterize the model’s strengths and limitations in recognizing novel, modified, and unrelated epitope sequences.

### 2.7 Reproducibility and data availability

Code and Data: The source code for the model, dataset and analyses was made publicly available on GitHub (include link).

https://github.com/cparejabarrueto/epitopes

Additional material and further details are available in supplementary material.

## 3 Results

Our research focused on analyzing molecular descriptors and assessing various machine learning algorithms when confronted with two different negative datasets, with the aim of evaluating how the selection of the negative datasets critically impacts the predictive performance and reliability of the models.

### 3.1 Dataset

A total of 256 continuous epitopes were obtained, associated with 10 antigens from: hemagglutinin, matrix protein 1 matrix protein 2, neuraminidase, non-structural protein, nucleoprotein, polymerase basic protein 1, polymerase basic protein 2, nuclear export protein 1, and the catalytic subunit of direct RNA polymerase.

As mentioned in the materials section, two sets of negative data were created for testing the ability of machine learning models to distinguish between positive and negative epitope examples. Negative set A comprises 1795 samples while negative set B consists of 4312 fragments.

### 3.2 Selecting relevant molecular descriptors

In our research, we aim to prevent the “curse of dimensionality” effect (Berisha *et al.* 2021), which occurs when the high dimensionality of data results in decreased model performance due to increased complexity and overfitting. To address this, we have developed a feature selection procedure to identify the most influential descriptors in each classification scenario.

**Figure 2:**
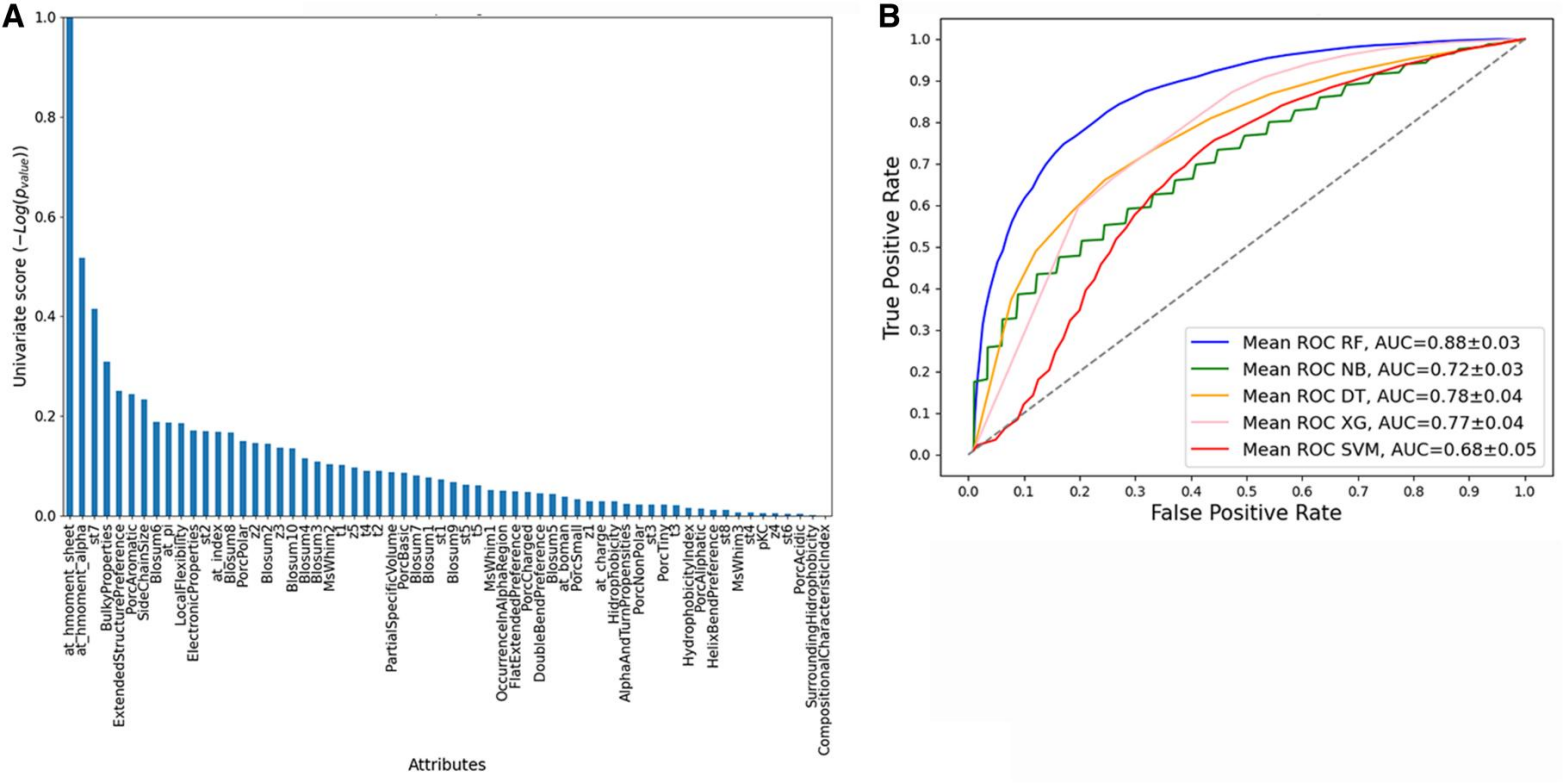
Comparative evaluation of classification models using sequence-derived descriptors in negative dataset A based on ANOVA feature selection. (A) Ranking of molecular descriptors selected by the ANOVA method. (B) ROC curves based on the 20 selected sequence-derived attributes, highlighting the superior performance of the Random Forest model (AUC = 0.87).

**Figure 3:**
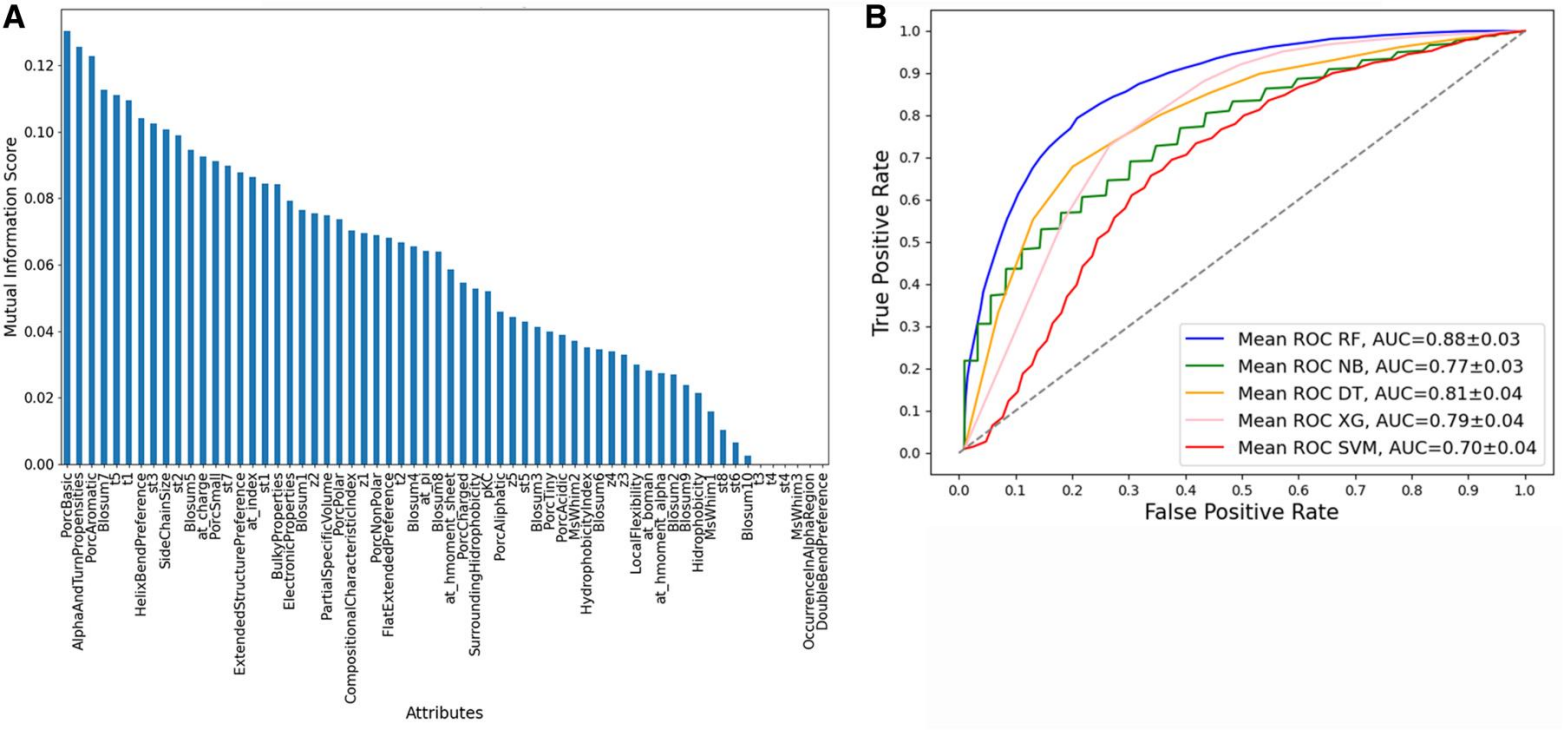
Comparative evaluation of classification models using sequence-derived descriptors in negative dataset A based on Mutual Information feature selection. (A) Ranking of molecular descriptors selected by Mutual Information analysis. (B) ROC curves based on the 20 selected sequence-derived attributes, highlighting the superior performance of the Random Forest model (AUC = 0.88)..

**Figure 4:**
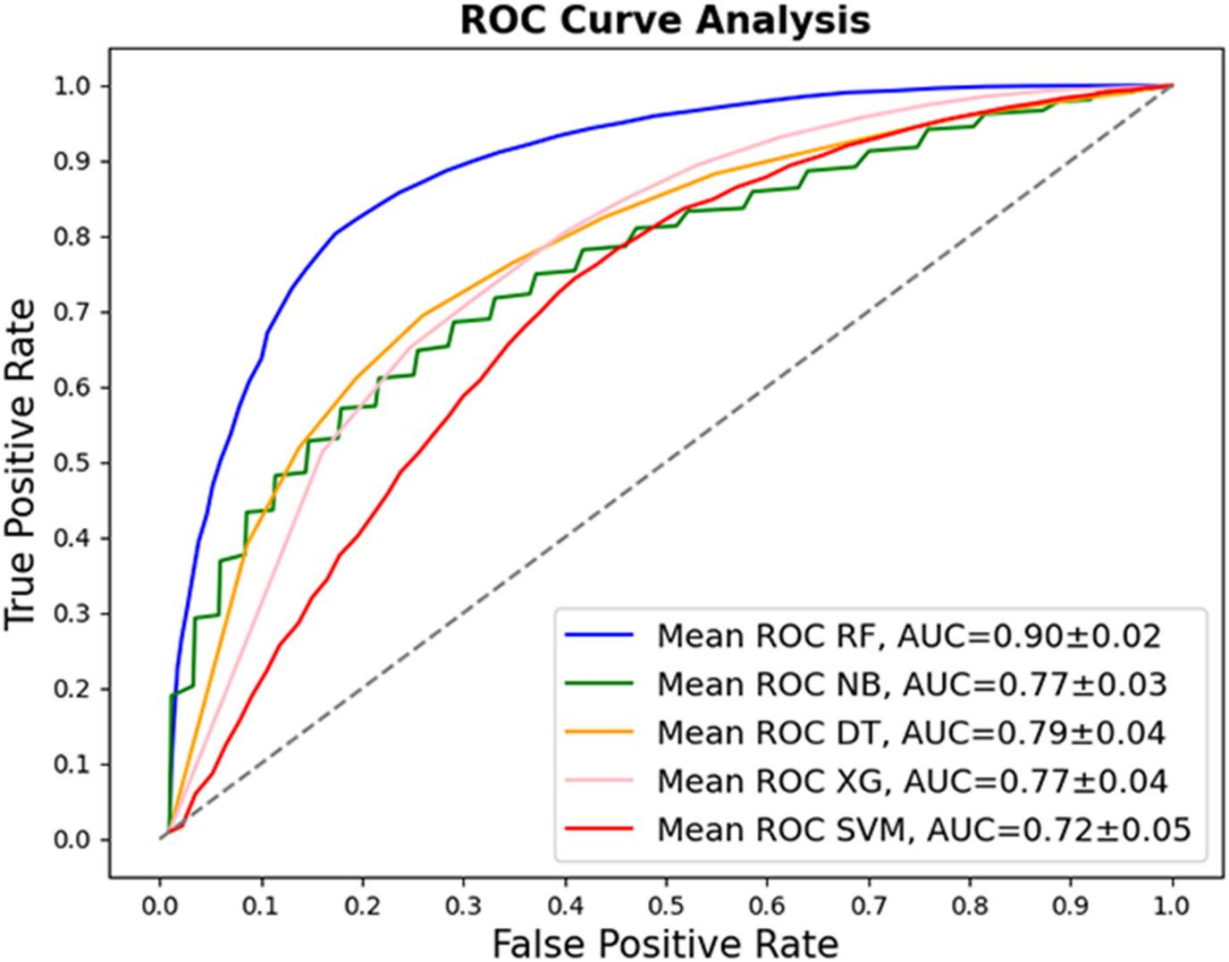
Comparative evaluation of classification models using sequence-derived descriptors in negative dataset A, using 32 sequence-derived descriptors selected by Mutual Information and ANOVA. (A) ROC curves for the classification models. The RF method displays the best performance with an AUC of 0.90.

**Figure 5:**
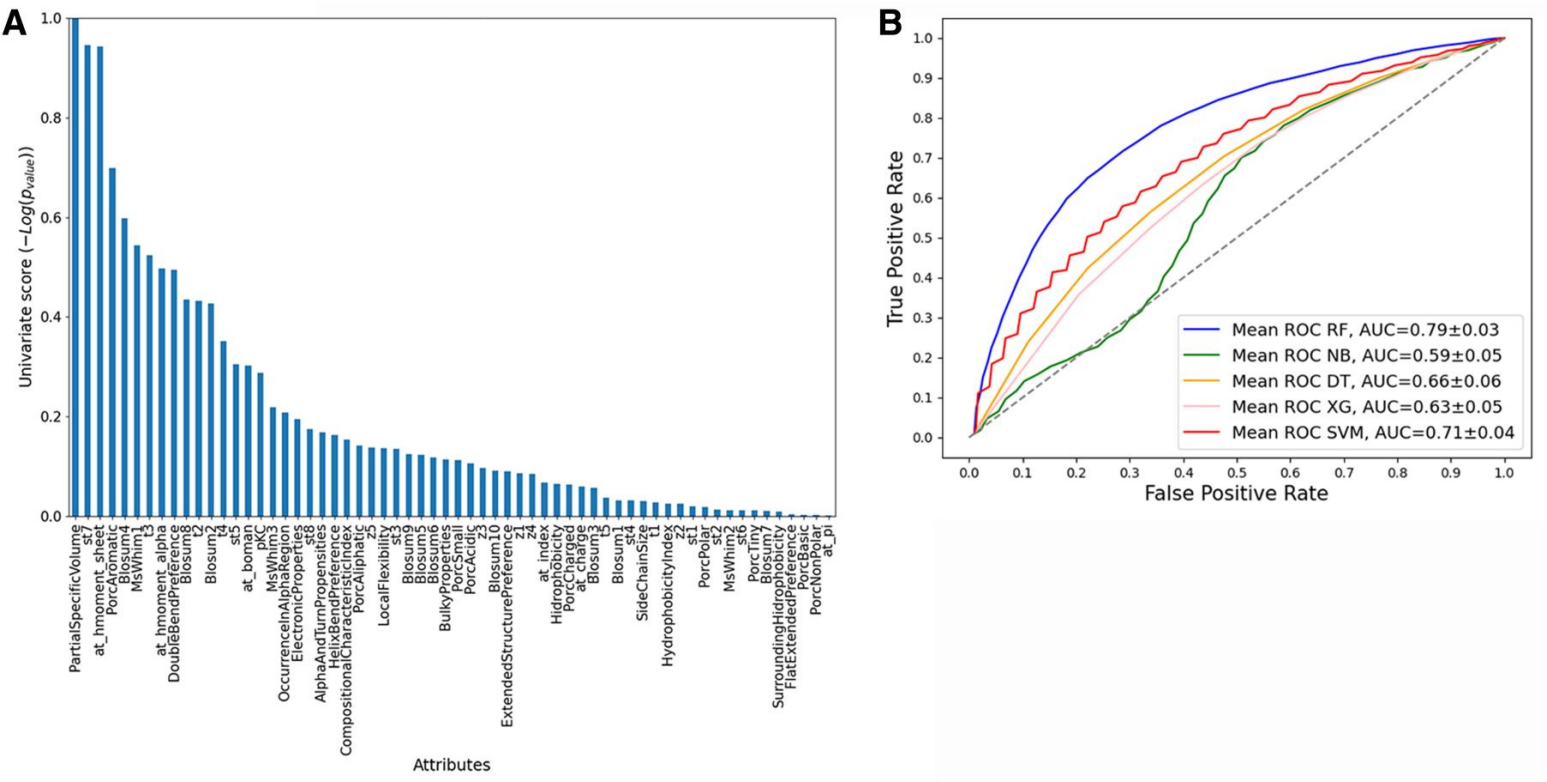
Comparative evaluation of classification models using sequence-derived descriptors in negative dataset B ANOVA. (A) Ranking of molecular descriptors by the ANOVA method. (B) ROC curves based on 20 sequence-derived attributes selected, highlighting the RF method for its superior performance (AUC of 0.81).

**Figure 6:**
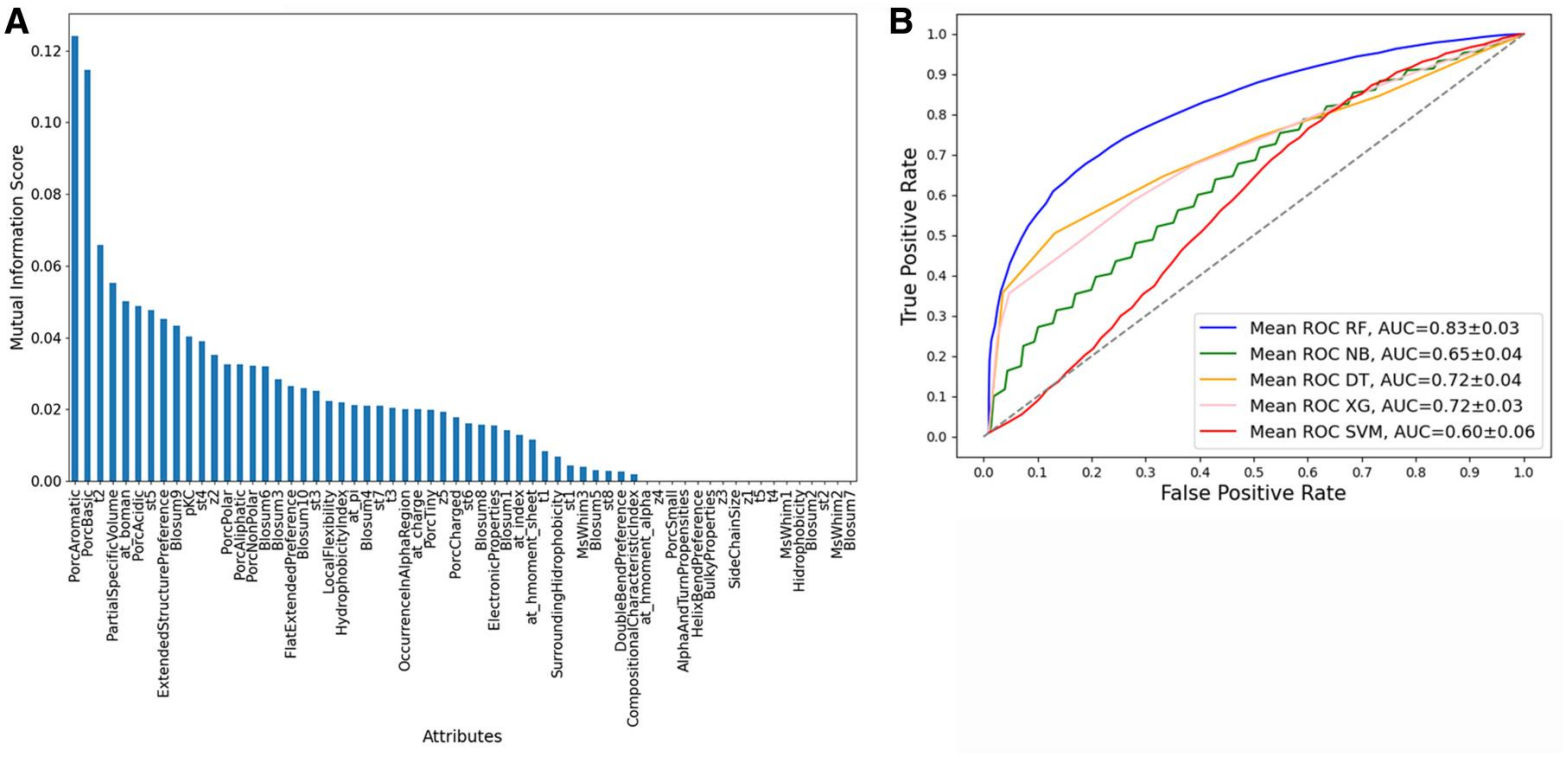
Comparative evaluation of classification models using sequence-derived descriptors in negative dataset B Mutual Information. (A) Ranking of molecular descriptors by Mutual Information method. (B) ROC curves based on 20 sequence-derived attributes selected, highlighting the RF method for its superior performance (AUC of 0.82).

The analysis of all 62 sequence-derived attributes was conducted employing both Mutual Information and ANOVA. Each of these selection functions yielded a ranked list, as illustrated in Figs 2A, 3A, 4, 5A, and 6A. For each analysis, the ranked lists were utilized to sequence the cumulative attribute evaluation. Our focus was on their discriminative power for differentiating between positive and negative epitope samples, considering both sets of negative samples in our analysis.

Following this ranking process, we examined the cumulative impact of adding these attributes one by one on model performance. The detailed results of this approach, using the Random Forest classifier, are presented in Annexes 1 and 2, available as supplementary data at *Bioinformatics Advances* online. We observed a consistent increase in model performance as more attributes were incorporated.

The cumulative plots for sequence-derived descriptors reveal that using the first-ranked attributes it is not possible to achieve a performance of >50%. However, when the number of attributes used in combination increased, the performance began to improve. In particular, integrating the top 20 ranked attributes in each case led to a plateau in performance, which led us to identify this number as a suitable cutoff for further evaluation in our study.

In our study, we chose 20 attributes each from the Mutual Information and ANOVA rankings, with eight attributes overlapping between the two. Consequently, a total of 32 unique attributes were incorporated for the combined analyses. The comprehensive list of these selected attributes is detailed in the supplementary material (see Annexes 3 and 4, available as supplementary data at *Bioinformatics Advances* online). These attributes were then employed in the evaluation of machine learning methodologies aimed at predicting new epitopes in novel influenza virus variants.

### 3.3 Evaluation of machine learning algorithms

We evaluated the SVM, NB, RF, XG, and DT machine learning algorithms using the selected sequence-derived attributes from ANOVA and Mutual Information method independently and in combination. As part of our research, we conducted independent evaluations of machine learning models using two separate negative datasets, A and B. To address potential issues with class imbalance resulting from the large number of negative examples (compared to the relatively small number of positive examples we analyzed), we followed a specific procedure. To mitigate class imbalance, random undersampling was applied to match the number of positive and negative samples (1:1 ratio) in each training iteration. This balancing procedure was repeated across 100 random resamplings to ensure metric stability.

The analysis performed for the negative dataset A applying the machine learning models using the 20 best attributes selected for ANOVA (Fig. 2A). The RF model performed better, with values of 79% accuracy, 80% recall, 80% precision, 80% F1-Score, and 59% MCC. The accuracy of the remaining models was 61% for NB, 66% SVM, 71% XG, and 71% DT (Table 1). While analysis using the 20 best attributes selected for Mutual Information (Fig. 3A), the RF model performed similar, with values of 79% accuracy, 82% recall, 78% precision, 79% F1-score, and 59% MCC. The accuracy of the remaining models was 67% for NB, 66% SVM, 73% XG, and 74% DT, Table 2.

**Table 1:** Performance of machine learning classifiers trained using the full set of sequence-derived descriptors.^a^

	Accuracy	Recall	Precision	F1 score	Matthew correlation
**RF**	0.79 ± 0.04	0.80 ± 0.04	0.80 ± 0.05	0.80 ± 0.04	0.59 ± 0.08
**NB**	0.61 ± 0.03	0.86 ± 0.02	0.58 ± 0.03	0.69 ± 0.02	0.29 ± 0.07
**DT**	0.71 ± 0.04	0.63 ± 0.09	0.77 ± 0.06	0.68 ± 0.06	0.44 ± 0.08
**XG**	0.71 ± 0.04	0.78 ± 0.06	0.69 ± 0.04	0.73 ± 0.04	0.43 ± 0.08
**SVM**	0.66 ± 0.05	0.74 ± 0.06	0.65 ± 0.05	0.69 ± 0.04	0.33 ± 0.09

a Metrics represent the mean ± SD obtained from five-fold cross-validation. The evaluated models include Random Forest (RF), Naïve Bayes (NB), Decision Tree (DT), XGBoost (XG), and Support Vector Machine (SVM).

**Table 2:** Performance of machine learning classifiers trained using the 20 most informative sequence-derived attributes selected by Mutual Information analysis for negative dataset A.^a^

	Accuracy	Recall	Precision	F1 score	Matthew correlation
**RF**	0.79 ± 0.03	0.82 ± 0.03	0.78 ± 0.04	0.79 ± 0.03	0.59 ± 0.06
**NB**	0.67 ± 0.03	0.86 ± 0.01	0.61 ± 0.03	0.71 ± 0.02	0.37 ± 0.06
**DT**	0.74 ± 0.04	0.71 ± 0.08	0.76 ± 0.06	0.73 ± 0.04	0.49 ± 0.07
**XG**	0.73 ± 0.04	0.82 ± 0.07	0.70 ± 0.05	0.75 ± 0.04	0.48 ± 0.08
**SVM**	0.66 ± 0.04	0.71 ± 0.04	0.63 ± 0.04	0.67 ± 0.03	0.32 ± 0.07

a Reported values correspond to the mean ± SD obtained from five-fold cross-validation.

The ROC curves for the classification models that utilized sequence-based descriptors show that the RF model performed significantly better than the other machine learning models evaluated with an AUC of 0.88 for ANOVA (Fig. 2B) and AUC 0.88 for Mutual Information feature selection approaches (Fig. 3B) analysis.

The analysis using the combination of the best attributes selected by ANOVA and Mutual Information showed that the best performance was achieved with the RF model, reaching 82% accuracy, 84% recall, 82% precision, 83 F1-score, and 0.65 for MCC. The accuracy of the remaining models was 67% for NB, 67% SVM, 71% XG, and 71% DT (Table 3). The ROC curves for the classification models that utilized sequence-based descriptors are presented in Fig. 4A. These show that the RF model performed significantly better than the other machine learning models evaluated with an AUC of 0.90.

**Table 3:** Performance comparison of machine learning classifiers using the combined feature set derived from sequence descriptors and feature selection procedures.^a^

	Accuracy	Recall	Precision	F1 score	Matthew correlation
**RF**	0.82 ± 0.03	0.84 ± 0.03	0.82 ± 0.04	0.83 ± 0.03	0.65 ± 0.06
**NB**	0.67 ± 0.04	0.82 ± 0.01	0.64 ± 0.04	0.72 ± 0.02	0.36 ± 0.07
**DT**	0.72 ± 0.04	0.66 ± 0.09	0.77 ± 0.06	0.70 ± 0.05	0.46 ± 0.08
**XG**	0.71 ± 0.04	0.76 ± 0.07	0.70 ± 0.05	0.73 ± 0.04	0.43 ± 0.09
**SVM**	0.67 ± 0.05	0.73 ± 0.05	0.66 ± 0.05	0.69 ± 0.04	0.34 ± 0.09

a Results are presented as mean ± SD from five-fold cross-validation.

The analysis performed for the negative dataset B, using the 20 best attributes selected for ANOVA (Fig. 5A), show the best model was RF with a 72% of accuracy, 73% recall, and 72% precision, 72% F1-Score, and 44% MCC (Table 4). The accuracy of the remaining models was 58% for NB, 61% SVM, 60% XG, and 62% DT. The analysis using the 20 best attributes selected for Mutual information show the better model was RF with 75% accuracy, 69% recall, 79% precision, 73% F1-Score, and 50% MCC (Table 5). The accuracy of the remaining models was 60% for NB, 56% SVM, 66% XG, and 68% for DT.

**Table 4:** Performance of machine learning classifiers evaluated using negative dataset B.^a^

	Accuracy	Recall	Precision	F1 score	Matthew correlation
**RF**	0.72 ± 0.04	0.73 ± 0.04	0.72 ± 0.04	0.72 ± 0.03	0.44 ± 0.07
**NB**	0.58 ± 0.03	0.90 ± 0.01	0.55 ± 0.02	0.68 ± 0.02	0.19 ± 0.08
**DT**	0.62 ± 0.05	0.56 ± 0.11	0.63 ± 0.06	0.59 ± 0.07	0.24 ± 0.10
**XG**	0.60 ± 0.04	0.63 ± 0.12	0.60 ± 0.04	0.61 ± 0.07	0.21 ± 0.09
**SVM**	0.61 ± 0.04	0.88 ± 0.04	0.58 ± 0.03	0.70 ± 0.02	0.26 ± 0.07

a Models were trained using the selected sequence-derived descriptors and evaluated by five-fold cross-validation. Values represent mean ± SD.

**Table 5:** Performance of machine learning classifiers trained using the selected feature subset and evaluated on Negative Dataset B using five-fold cross-validation.^a^

	Accuracy	Recall	Precision	F1 score	Matthew correlation
**RF**	0.75 ± 0.03	0.69 ± 0.04	0.79 ± 0.05	0.73 ± 0.03	0.50 ± 0.07
**NB**	0.60 ± 0.04	0.64 ± 0.05	0.60 ± 0.04	0.62 ± 0.04	0.20 ± 0.08
**DT**	0.68 ± 0.05	0.59 ± 0.09	0.73 ± 0.07	0.65 ± 0.06	0.37 ± 0.09
**XG**	0.66 ± 0.04	0.64 ± 0.10	0.69 ± 0.09	0.65 ± 0.04	0.33 ± 0.08
**SVM**	0.56 ± 0.05	0.49 ± 0.11	0.56 ± 0.11	0.52 ± 0.11	0.11 ± 0.09

a Metrics represent mean ± SD across validation folds.

The ROC curves for the classification models that utilized sequence-based descriptors show that the RF model performed significantly better than the other machine learning models evaluated with an AUC of 0.79 for ANOVA (Fig. 5B) and AUC 0.83 Mutual Information (Fig. 6B) analysis.

**Figure 7:**
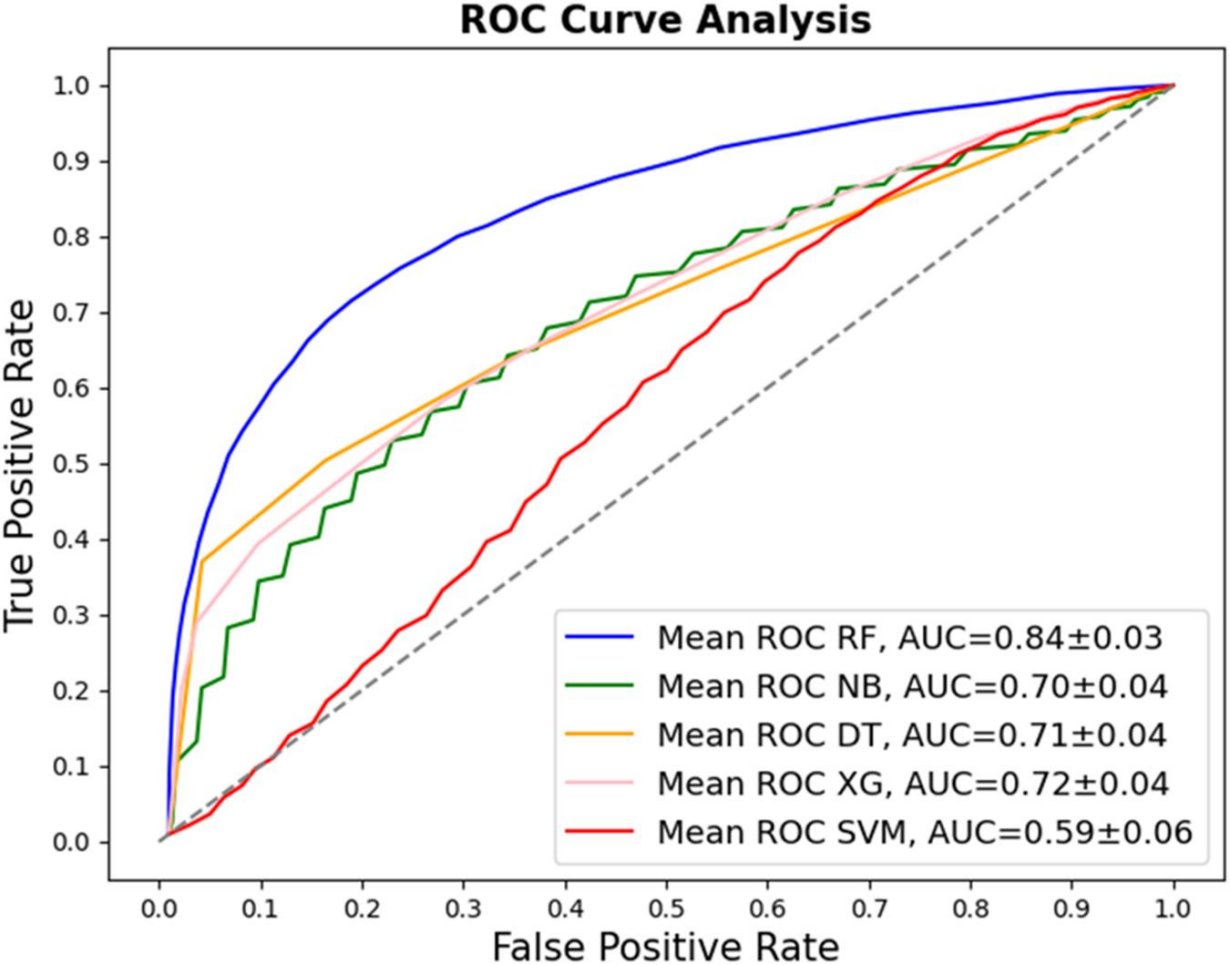
Comparative evaluation of classification models using sequence-derived descriptors in negative dataset B, using 32 sequence-derived descriptors selected by Mutual Information and ANOVA. (A) ROC curves for the classification models. The RF method displays the best performance with an AUC of 0.80.

In this case, the analysis using the combination of the best attributes selected by ANOVA and Mutual Information showed that the best performance was achieved with the RF, reaching 77% accuracy, 74% recall, 79% precision, 76% F1-score, and 54% MCC. The accuracy of the remaining models was 65% for NB, 55% SVM, 66% XG, and 67% DT (Table 6). The ROC curves for the classification models that utilize sequence-based descriptors are presented in Fig. 7A. These show that the RF model performed significantly better than the other machine learning models evaluated with an AUC of 0.84.

**Table 6:** Final performance comparison of machine learning classifiers using the optimized feature subset and training configuration.^a^

	Accuracy	Recall	Precision	F1 score	Matthew correlation
**RF**	0.77 ± 0.04	0.74 ± 0.04	0.79 ± 0.05	0.76 ± 0.04	0.54 ± 0.07
**NB**	0.65 ± 0.05	0.68 ± 0.06	0.64 ± 0.05	0.66 ± 0.04	0.29 ± 0.10
**DT**	0.67 ± 0.05	0.59 ± 0.09	0.71 ± 0.08	0.64 ± 0.06	0.35 ± 0.10
**XG**	0.66 ± 0.04	0.65 ± 0.08	0.67 ± 0.07	0.65 ± 0.04	0.32 ± 0.09
**SVM**	0.55 ± 0.05	0.48 ± 0.09	0.56 ± 0.08	0.52 ± 0.08	0.10 ± 0.09

a Metrics represent mean ± SD obtained from five-fold cross-validation.

To further evaluate the robustness of our model in recognizing variations of known epitopes, we tested its performance on a set of linear BCEs derived from Influenza A, artificially mutated at increasing levels (10%, 20%, 30%, 50%, and 70% of mutation). The results revealed a progressive decline in predictive performance as the degree of mutation increased (Table 7). At 10% mutation, the model maintained high accuracy (83%) and recall (100%), suggesting strong tolerance to minor sequence alterations. However, as mutations reached 30% and beyond, accuracy and F1-score dropped substantially, with the lowest performance observed at 70% mutation (accuracy: 33%, F1-score: 0.50). These results demonstrate the model’s ability to recognize mildly altered epitopes, while highlighting its reduced sensitivity to extensively mutated sequences.

**Table 7:** Performance analysis of the model evaluated with different test epitope sets, including sequences subjected to increasing mutation levels, inverted sequences, and unrelated Dengue virus epitopes.^a^

Test condition	Accuracy	Precision	Recall	F1-score
**Mutation level - 10%**	83	83	100	91
**Mutation level - 20%**	77	77	100	87
**Mutation level - 30%**	64	64	100	78
**Mutation level - 50%**	46	46	100	63
**Mutation level - 70%**	33	33	100	50
**Dengue epitope**	2.6	100	2.6	5
**Inverted positives**	84.6	100	84.6	92

a Results are reported as percentages of accuracy, precision, recall, and F1-score.

Sensitivity analysis revealed that classification performance depends on peptide length when generating artificial negatives (Annex 15, available as supplementary data at *Bioinformatics Advances* online). While 12-mer windows yielded higher predictive metrics, performance progressively decreased for 14-mers and 16-mers. This pattern is consistent with increased heterogeneity introduced by longer windows, which may incorporate residues not directly contributing to epitope-specific physicochemical signatures. For consistency with the primary benchmark design and biological representativeness, we retained 14 amino acids for the main analyses.

To test the model’s ability to generalize to unrelated viral epitopes, we evaluated it using BCEs derived from the Dengue virus. The model achieved very low recall (2.6%) and accuracy (2.6%), despite a perfect precision of 100% (Table 7). This result indicates that the model is highly conservative when facing antigenically unrelated sequences: it avoids false positives but fails to identify most true epitopes. Such performance suggests that the features learned from Influenza A epitopes do not effectively translate to epitopes from Dengue virus, underlining the antigenic specificity of the trained model.

To assess the model’s sensitivity to sequence directionality, we tested it using a set of known positive epitopes that were reversed (amino acid order inverted). The model retained high performance, with 84.6% accuracy and 100% precision (Table 7), indicating that it is still capable of identifying immunogenic potential in sequences where linear order is altered. This suggests that the model recognizes local residue patterns or physicochemical profiles that persist regardless of sequence orientation.

### 3.4 CD-HIT threshold impacts on classification performance

To assess the influence of sequence redundancy on model performance, we trained and evaluated several classifiers across four CD-HIT identity thresholds (95%, 90%, 85%, and 80%, Annex 7, available as supplementary data at *Bioinformatics Advances* online) using distinct feature selection strategies (ANOVA, Mutual Information, and combined). Random Forest consistently achieved the best overall performance across all thresholds and selection methods. Notably, the highest F1-score was observed at the 85% threshold using the full feature set (F1 = 0.80 ± 0.05), along with a balanced precision (0.81 ± 0.06) and recall (0.79 ± 0.05), highlighting this threshold as the optimal trade-off between diversity and sample size, Annexes 8–11, available as supplementary data at *Bioinformatics Advances* online.

In contrast, the 80% threshold, while increasing sequence diversity, led to a slight decline in model performance (RF F1 = 0.72 ± 0.04), possibly due to the reduced number of training samples (*n* = 148 epitopes). Similarly, Naive Bayes and Decision Trees exhibited moderate declines in metrics such as accuracy and MCC at lower thresholds. Among feature selection strategies, combining ANOVA and Mutual Information generally improved classifier robustness, particularly at intermediate thresholds (90%–85%), Annexes 8–11, available as supplementary data at *Bioinformatics Advances* online.

Interestingly, performance with Support Vector Machines remained relatively lower across all thresholds, with F1-scores consistently below 0.65, suggesting that this classifier may be more sensitive to training data variability. Overall, these results demonstrate that filtering epitope datasets with CD-HIT at moderate identity thresholds (85%–90%) strikes a balance between sequence diversity and model performance, enabling robust classification without excessive redundancy.

### 3.5 Comparative performance against state-of-the-art epitope prediction methods

To contextualize the performance of our model, we conducted a comparative evaluation against several state-of-the-art epitope prediction tools. This included both classical machine-learning–based predictors and recent deep-learning approaches. By applying these external tools to our curated dataset, we quantified their predictive accuracy and directly contrasted their performance with that of our proposed model. After reducing sequence redundancy with CD-HIT at an 85% identity threshold, the positive and negative datasets were balanced to 152 epitopes per class and used to perform tests with the different software.

### 3.6 BepiPred-3.0

According to the BepiPred-3.0 publication, the recommended default classification threshold is 0.1512 (Clifford *et al.* 2022). Using this value, we evaluated the binary prediction performance of BepiPred-3.0 on both our positive and negative datasets balanced.

We found that 92.6% of residues in the negative set exceeded the 0.1512 threshold, indicating that BepiPred-3.0 assigns a large majority of non-epitopic positions to the epitope class. Similarly, 97.4% of residues in the positive dataset surpassed the same threshold. Thus, although the positive distribution is slightly shifted toward higher scores, the degree of overlap between positive and negative residues is extremely high (Table 8).

**Table 8:** Comparative performance of state-of-the-art B-cell epitope prediction tools on our balanced benchmark dataset (152 positives and 152 negatives).

Metric	BepiPred-3.0^a^	iBCE-EL	EpitopeVec
**Granularity**	Residue-level	Peptide-level	Peptide-level
**TP**		68	65
FN		84	87
**TN**		107	129
**FP**		45	23
**Sensitivity (TPR)**	0.974	0.447	0.428
**Specificity (TNR)**	0.074	0.704	0.849
**False positive rate (FPR)**	0.926	0.296	0.151
**Accuracy**	∼0.535	0.576	0.638
**Precision (PPV)**	0.627	0.602	0.739
**Negative predictive value (NPV)**	0.200	0.560	0.597
**Balanced accuracy**	0.524	0.576	0.638
**MCC**	∼0	0.157	0.304
**Main weakness**	Extremely high FPR	Low sensitivity	Misses many positives
**Main strength**	Very high TPR	Moderate balance	Best MCC/best specificity
**AUC (ROC)** ^b^	0.55	0.69	0.68

a BepiPred-3.0 metrics reflect residue-level classification using the official threshold (0.1512) and are therefore not directly comparable at the TP/TN level. However, sensitivity, specificity, FPR and balanced accuracy remain valid for high-level comparison.

b Our best-performing model (Random Forest with 32 descriptors) achieved an AUC of ∼0.90 (0.896).

These results show that BepiPred-3.0, at its official classification threshold, provides almost no discriminative power on our dataset, yielding a very high false-positive rate. This highlights that BepiPred-3.0 is better used as a continuous ranking model rather than as a threshold-based classifier, and it underscores the need for alternative machine-learning approaches capable of effectively separating epitope and non-epitope regions in our sequences.

### 3.7 iBCE-EL

Using the iBCE-EL (Manavalan *et al.* 2018) web server in peptide mode (PIP-EL), we evaluated its performance on our curated benchmark, which comprises 152 experimentally validated BCEs and 152 experimentally verified non-BCEs. Out of the 152 positive peptides, 68 were correctly classified as BCEs (TP), whereas 84 were misclassified as non-BCEs (FN). For the negative set, 107 peptides were correctly predicted as non-BCEs (TN) and 45 were incorrectly predicted as BCEs (FP). This corresponds to a sensitivity of 0.45, a specificity of 0.70, an overall accuracy of 0.58 and an MCC of 0.16, indicating only modest discriminative power of iBCE-EL on our dataset (Table 8).

### 3.8 EpitopeVec

EpitopeVec (Bahai *et al.* 2021) displayed moderate classification performance on our balanced benchmark of 152 experimentally validated epitopes and 152 non-epitopes. The method correctly identified 65 positive peptides (sensitivity = 0.428), indicating that fewer than half of the true epitopes were recognized by the model. In contrast, specificity was substantially higher (0.849), with only 23 negative peptides misclassified as epitopes (FPR = 0.151). EpitopeVec also achieved the highest precision among the evaluated tools (PPV = 0.739), reflecting a relatively low rate of false positives. Overall accuracy reached 0.638, and the Matthews correlation coefficient (MCC = 0.304) further indicated moderate discriminative power. Together, these results show that while EpitopeVec is effective at avoiding false positives, it fails to detect a large proportion of true epitopes, highlighting a strong bias toward conservative predictions and limited generalization to the full diversity of experimentally validated epitope sequences included in our dataset (Table 8).

A comparative analysis of BepiPred-3.0, iBCE-EL, and EpitopeVec revealed limitations in their ability to discriminate epitopes from non-epitopes in our curated Influenza A dataset. Using its official threshold (0.1512), BepiPred-3.0 achieved high sensitivity (0.974) but extremely low specificity (0.074), resulting in a false-positive rate of 0.926 and a balanced accuracy close to random performance. iBCE-EL showed improved specificity (0.704) but considerably lower sensitivity (0.447), yielding modest overall accuracy and a low MCC (0.157). EpitopeVec exhibited a different failure mode: although it produced high scores for canonical epitope hotspots, ∼16% of experimentally validated epitopes received scores <0.60, indicating poor generalization beyond classical motifs. Collectively, these results demonstrate that existing state-of-the-art tools perform suboptimally on our benchmark and justify the development of alternative machine-learning approaches with improved discriminative power.

**Figure 8:**
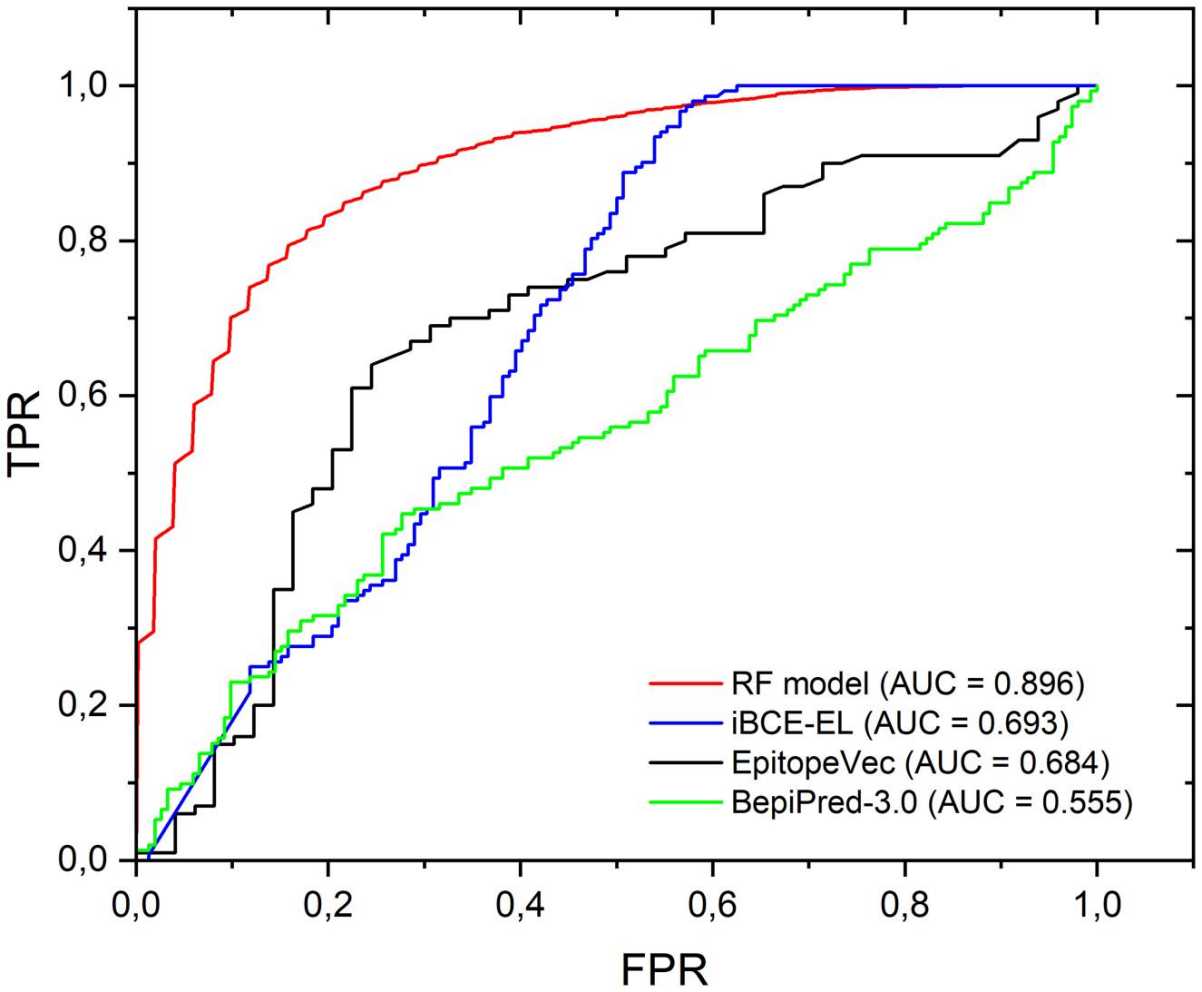
Receiver operating characteristic (ROC) curves comparing the proposed Random Forest model with state-of-the-art B-cell epitope prediction tools. The RF model achieved the highest discriminative performance (AUC = 0.896), outperforming iBCE-EL (0.693), EpitopeVec (0.684), and BepiPred-3.0 (0.555).

To provide a threshold-independent comparison of predictive performance, receiver operating characteristic (ROC) curves were generated for the proposed Random Forest model and benchmarked against widely used BCE prediction tools, including BepiPred-3.0, iBCE-EL, and EpitopeVec (Fig. 8).

The Random Forest model showed the highest discriminative ability, achieving a mean AUC of 0.896 (∼0.9) across 100 independent repetitions, substantially outperforming all reference methods. In contrast, iBCE-EL and EpitopeVec displayed moderate performance, with AUC values of 0.693 and 0.684, respectively, whereas BepiPred-3.0 showed near-random classification behavior (AUC = 0.555).

The ROC profile of the proposed model demonstrated consistently higher true positive rates across most false positive rate thresholds, indicating improved sensitivity-specificity balance and stronger ranking capability for distinguishing validated epitopes from non-epitope sequences.

In addition to these recently developed tools, we also examined several classical epitope predictors such as ABCpred (Saha and Raghava 2006), BcePred (Saha and Raghava 2004), and VaxiJen (Doytchinova and Flower 2007). However, these methods rely on outdated training sets and simplified algorithms that are no longer competitive with current approaches and therefore were not included in the main comparative analysis. Likewise, other available tools including NetMHCpan (Reynisson *et al.* 2020), which focuses on T-cell epitope presentation, and paratope-based frameworks such as EPP (Epitope–Paratope Predictor) (Li *et al.* 2025) operate under fundamentally different predictive principles and are not directly comparable to linear BCE classifiers. This category also includes recent, sophisticated models like ProtBERT/ProtXLNet, which leverage transformer architectures pre-trained on protein sequences to predict T-cell receptor (TCR)-epitope binding specificity (Khan *et al.* 2023).

For these reasons, our benchmarking concentrated on BepiPred-3.0, iBCE-EL, and EpitopeVec, which represent the most relevant state-of-the-art methods for peptide-level BCE prediction using sequence information.

## 4 Discussion

The accurate and high-throughput prediction of BCEs remains a bottleneck in vaccinology, particularly for highly variable pathogens such as Influenza A. Our study presents a machine learning framework that effectively addresses two core limitations of current generalized epitope prediction tools: the lack of biological relevance in negative datasets and the use of black-box models that obscure the underlying physicochemical determinants of immunogenicity. The overall predictive performance, as demonstrated by our results, is critically dependent on both the biological representativeness of the training data and the physicochemical characterization of the sequences. Specifically, the Random Forest model consistently exhibited the highest performance metrics, validating the value of tailored, pathogen-specific approaches built on interpretable features to overcome the weaknesses of current state of the art methodologies.

A key contribution of this work is the explicit evaluation of how negative dataset construction shapes model performance. Models trained on real, experimentally confirmed non-epitopes demonstrated superior discrimination (82% accuracy) compared to those trained on arbitrary artificial fragments. This reveals that using artificial negative examples can inflate performance metrics at the cost of poor generalization. Our results highlight the necessity of rigorous negative set curation that reflects genuine biological non-recognition, an aspect frequently overlooked in epitope prediction workflows.

Across all experiments, Random Forest consistently outperformed SVM, NB, DT, and XGBoost, highlighting the ability of tree-based ensembles to capture nonlinear relationships between physicochemical properties and immunogenicity. Feature selection analyses revealed that individual descriptors have limited predictive value, but their cumulative integration, particularly the top 20–30 ranked features, substantially enhances classification performance. These findings reinforce the idea that epitope immunogenicity emerges from composite physicochemical signatures rather than isolated residue-level properties. Importantly, the selected descriptors provide interpretable biological insights, contrasting with high-dimensional protein embedding models that function as black boxes.

Benchmarking our model against widely used general-purpose predictors such as BepiPred-3.0, iBCE-EL, and EpitopeVec indicated reduced discriminative performance on our Influenza A-specific benchmark, highlighting the challenges of applying broadly trained predictors to pathogen-specific datasets. BepiPred-3.0 functioned more effectively as a continuous ranking system than as a binary classifier, producing a high false-positive rate at its recommended threshold. This behavior likely reflects the design goal of the method, which prioritizes sensitivity in identifying potential epitope regions rather than strict binary classification. As a result, when applied to short peptide classification tasks such as those used in this study, the method tends to produce a high number of positive predictions.

While EpitopeVec and iBCE-EL showed improved specificity, they exhibited moderate to low sensitivity, failing to detect a substantial proportion of experimentally validated epitopes. Although iBCE-EL achieved a higher true positive rate, this was accompanied by an increased number of false positive predictions, leading to lower balanced accuracy and MCC values compared with the Random Forest model. This illustrates the common trade-off between sensitivity and overall classification balance in epitope prediction models. It is important to note that several widely used predictors, including BepiPred-3.0, were originally designed for identifying potential epitope regions along protein sequences rather than performing strict peptide-level binary classification. As a consequence, these methods tend to prioritize sensitivity and may predict a large fraction of residues or peptides as epitope candidates. When applied to short peptide classification tasks such as those used in this study, this behavior can result in an increased number of positive predictions and reduced specificity.

In contrast, our descriptor-based RF model achieved a superior balance (MCC = 0.65), demonstrating that pathogen-specific approaches, when coupled with rigorous feature engineering, can offer enhanced discriminatory power for applied tasks like epitope-driven vaccine design.

Evaluation with mutated, reversed, and cross-viral epitopes further clarified the model’s strengths and boundaries. The performance analysis on external test sets provides biological evidence regarding what the model has learned. The rapid decline in predictive performance beyond 30% mutation suggests that the model has learned sequence patterns closely associated with Influenza A epitope characteristics. These patterns likely include local physicochemical properties and residue composition features captured by the sequence-derived descriptors used for model training. As mutations accumulate, these signals become progressively disrupted, leading to a decrease in classification accuracy.

The reduced performance observed on Dengue virus epitopes highlights a limitation of the proposed approach. While the model performs well for Influenza A, its predictive capacity decreases when applied to antigenically distinct pathogens. This behavior reflects a trade-off inherent to pathogen-specific machine learning models, where increased specialization may come at the cost of reduced generalizability. In this context, pathogen-focused predictors should be viewed as complementary to general-purpose tools rather than universal replacements. Together, these external validation tests confirm that our model has successfully captured robust and biologically meaningful characteristics pertinent to BCE prediction in the context of Influenza A virus.

Our CD-HIT evaluation demonstrated that intermediate redundancy thresholds (85%–90%) provide the optimal trade-off between diversity and sample size. Too little redundancy reduction inflates performance through memorization of highly similar sequences, whereas overly aggressive filtering reduces the amount of training data. These findings support the broader consensus that redundancy control parameters are not merely technical details but central determinants of model generalization.

While recent deep-learning approaches leveraging transformer embeddings (e.g. ProtBert, ESM) have shown strong performance, they require large training sets and offer limited interpretability. Our results demonstrate that interpretable, descriptor-based models remain competitive when paired with rigorous feature selection and biologically grounded dataset construction. Furthermore, pathogen-specific models such as ours may offer superior precision for vaccine-design pipelines, particularly when rapid updates are required for evolving viruses such as Influenza A.

Our work positions interpretable, pathogen-specific models as a competitive and complementary alternative to complex, general-purpose deep learning models. For rapidly evolving viruses like Influenza A, where agile vaccine updates are critical, the capacity to rapidly and accurately identify candidate epitopes with a clear physicochemical rationale is a tangible advantage. The presented strategy, emphasizing negative data quality, intelligent descriptor selection, and rigorous generalization testing, provides a robust framework that can accelerate epitope discovery and prioritize candidates for peptide-based vaccine development or immunogen redesign.

A limitation of this study is its exclusive focus on linear epitopes, leaving aside conformational epitopes that are crucial for many neutralizing antibody responses. Future work must integrate structural descriptors or 3D model information to address this challenge. Furthermore, we will explore building hybrid models that combine the rich representational power of PLM embeddings (e.g. from ESM or ProtBert) with the interpretability of our molecular descriptors. This integration may bridge data-driven representation learning and mechanistic understanding, potentially improving generalization across related viral families while retaining model transparency.

## 5 Conclusions

Our analysis of existing methodologies reveals that some have lower performance compared to ours. This discrepancy often stems from their pursuit of a “general” approach, applicable to epitope detection across various pathogens (viruses, bacteria, fungi). Such methods typically employ broad training sets. In contrast, our method is specifically trained and fine-tuned for a certain type of virus, which significantly boosts its efficacy in identifying epitopes in new strains of influenza A.

In turn, we can conclude that the use of molecular descriptors based on physicochemical properties that can be extracted from the amino acid sequence provides enough information to generate reliable predictive models. The implementation of the correct molecular descriptors in epitope prediction not only optimizes the accuracy of the predictions.

We can conclude that the careful selection and evaluation of negative datasets become an indispensable step in enhancing the predictive capability and practical utility of machine learning models in the identification of epitopes. This allows us to accelerates the process of identifying potentially immunogenic epitopes in emerging viruses, a critical advancement in the era of rapid viral mutations and global pandemics.

## Supplementary Material

vbag127_Supplementary_Data

## Data Availability

The datasets used for model training and testing were obtained from The Immune Epitope Database (IEDB, https://www.iedb.org/). All scripts and processed datasets generated during the current study are publicly available at https://github.com/cparejabarrueto/epitopes.
