# Supplementary material for: The impact of biologically relevant negative samples on machine learning-based B-cell epitope prediction for Influenza A
Source: Bioinform Adv. 2026 May 4;6(1):vbag127. doi: 10.1093/bioadv/vbag127 (PMC13197123; doi:10.1093/bioadv/vbag127)
Supplement: vbag127_Supplementary_Data [file vbag127_supplementary_data.docx]

**Annex 1: Performance of 62 accumulated attributes ranked with Anova and Mutual Information, for the Negative Dataset A.**

**
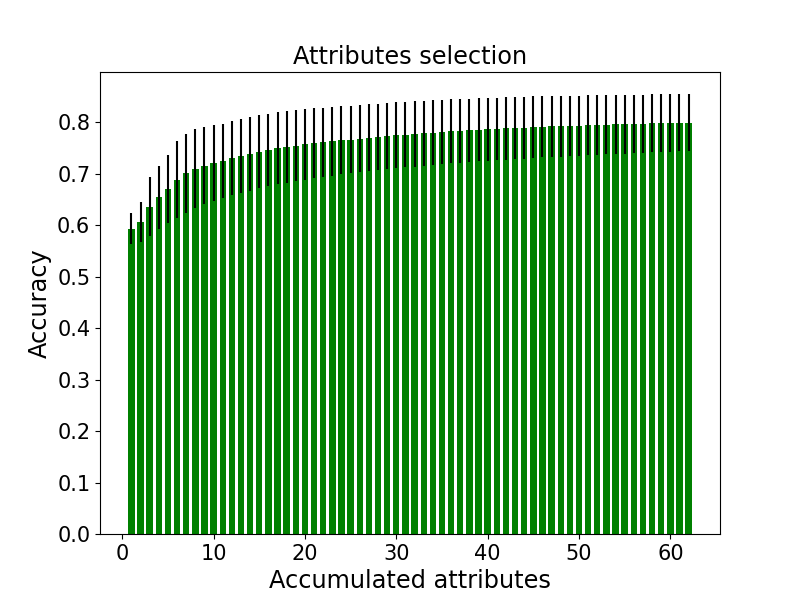

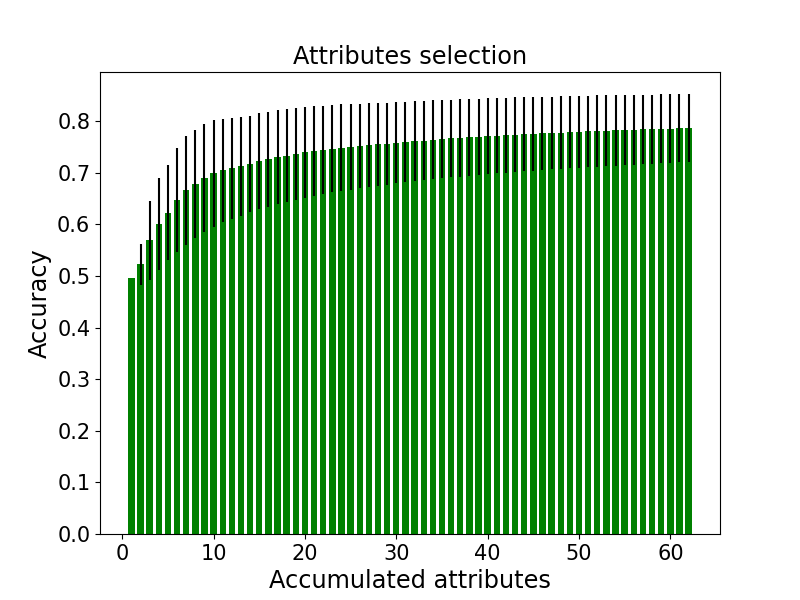
**

Mutual information

Anova

**Annex 2: Performance of 62 accumulated attributes ranked with Anova and Mutual Information, for the Negative Dataset B.**

**
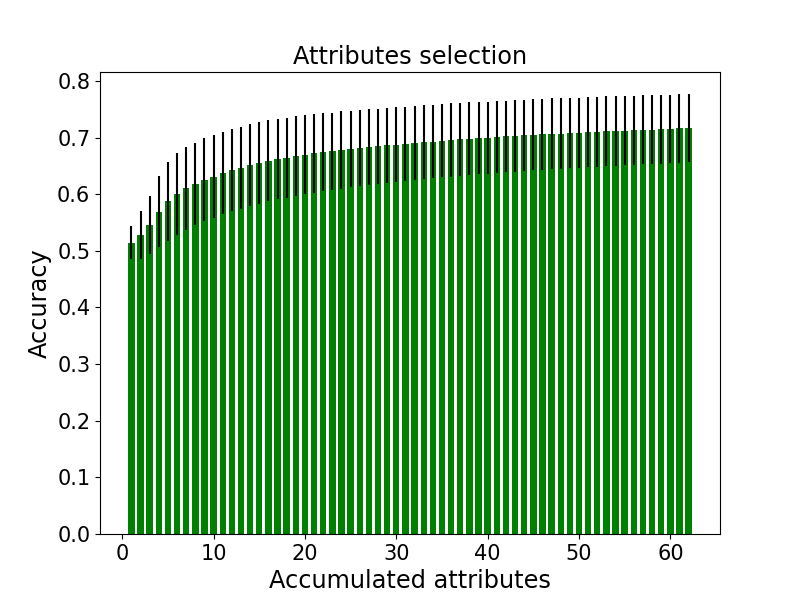
**

**Mutual information**

**
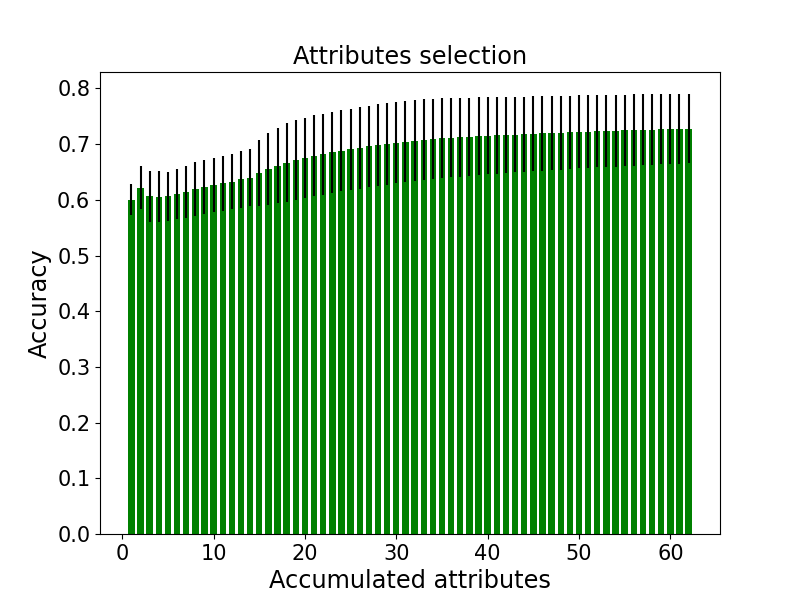
**

**Anova**

**Annex 3:** **list of these selected attributes for the negative data set A.** The name of the attributes is based in the software Peptide.

Selection of best by ANOVA (20):

at_hmoment_sheet, at_hmoment_alpha, st7, BulkyProperties, ExtendedStructurePreference, PorcAromatic, SideChainSize, Blosum6, at_pi, LocalFlexibility, ElectronicProperties, st2, at_index, Blosum8, PorcPolar, z2, Blosum2, z3, Blosum10, and Blosum4.

Mutual Information (20):

PorcBasic, AlphaAndTurnPropensities, PorcAromatic, Blosum7, t5, t1, HelixBendPreference, st3, SideChainSize, st2, Blosum5, at_charge, PorcSmall, st7, ExtendedStructurePreference, at_index, st1, BulkyProperties, ElectronicProperties, and Blosum1.

Combination of selected attributes (32):

at_hmoment_sheet, at_hmoment_alpha, st7, BulkyProperties,

ExtendedStructurePreference, PorcAromatic, SideChainSize, Blosum6, at_pi,

LocalFlexibility, ElectronicProperties, st2, at_index, Blosum8, PorcPolar, z2, Blosum2, z3, Blosum10, Blosum4, PorcBasic, AlphaAndTurnPropensities, Blosum7, t5, t1,

HelixBendPreference, st3, Blosum5, at_charge, PorcSmall, st1, and Blosum1.

**Annex 4: list of these selected attributes for the negative data set B. T**he name of the attributes is based in the software Peptide.

Selection of best by ANOVA (20):

PartialSpecificVolume, st7, at_hmoment_sheet, PorcAromatic, Blosum4, MsWhim1, t3, at_hmoment_alpha, DoubleBendPreference, Blosum8, t2, Blosum2, t4, st5, at_boman, pKC, MsWhim3, OccurrenceInAlphaRegion, ElectronicProperties, and st8.

Mutual Information (20):

PorcAromatic, PorcBasic, t2, PartialSpecificVolume, at_boman, PorcAcidic, st5, ExtendedStructurePreference, Blosum9, pKC, st4, z2, PorcPolar, PorcAliphatic, PorcNonPolar, Blosum6, Blosum3, FlatExtendedPreference, Blosum10, st3

Combination of selected attributes (34):

PartialSpecificVolume, st7, at_hmoment_sheet, PorcAromatic, Blosum4, MsWhim1, t3, at_hmoment_alpha, DoubleBendPreference, Blosum8, t2, Blosum2, t4, st5, at_boman, pKC, MsWhim3, OccurrenceInAlphaRegion, ElectronicProperties, st8, PorcBasic, PorcAcidic, ExtendedStructurePreference, Blosum9, st4, z2, PorcPolar, PorcAliphatic, PorcNonPolar, Blosum6, Blosum3, FlatExtendedPreference, Blosum10, st3

**Annex 5: configuration of the used software**

For feature selection the used algorithms were:

ANOVA:

Using SelectKBest utility in scikit-learn were select features according to the *f_classif* function and *k=“all”* highest scores. The *f_classif* function use the ANOVA F-value between features for classification tasks.

Mutual Information:

Using SelectKBest utility in scikit-learn were select features according to the *mutual_info_classif* function and *k=“all”* highest scores. The *mutual_info_classif* function relies on nonparametric methods based on entropy estimation from k-nearest neighbors distances.

**Annex 6**: Description of the attributes calculated using the **Peptides** package in R. These attributes are useful in peptide characterization, including structural, electronic, and physicochemical properties.

1. **at_hmoment_sheet** - Hydrophobic moment for beta-sheet structures, which quantifies the amphiphilicity of the sequence in a beta-sheet conformation.
2. **at_hmoment_alpha** - Hydrophobic moment for alpha-helical structures, indicating amphiphilicity in an alpha-helical conformation.
3. **st7** - Possibly a descriptor related to steric properties of amino acids (based on the **stScales** indices).
4. **BulkyProperties** - Measures the steric bulk of amino acid side chains, impacting folding and interaction properties.
5. **SideChainSize** - Represents the size of the amino acid side chains, influencing protein folding and interactions.
6. **ExtendedStructurePreference** - Indicates the tendency of an amino acid to adopt an extended conformation (e.g., in beta-sheets).
7. **ElectronicProperties** - Describes the electronic characteristics of amino acids, including charge and dipole interactions.
8. **st2** - Likely another steric property from **stScales**, representing molecular volume.
9. **PorcAromatic** - Percentage of aromatic amino acids (F, Y, W) in the sequence.
10. **at_pi** - Theoretical isoelectric point (pI) of the peptide, the pH at which the net charge is zero.
11. **PorcPolar** - Percentage of polar amino acids in the sequence.
12. **z2** - Z- scale descriptor related to steric bulk and polarizability of amino acids.
13. **Blosum8** - A numerical representation derived from the BLOSUM62 matrix, capturing amino acid substitution patterns.
14. **at_index** - Aliphatic index, which measures the relative volume occupied by aliphatic side chains (A, I, L, V).
15. **z3** - Z-scale descriptor associated with electronic properties (polarity/charge).
16. **LocalFlexibility** - Reflects the flexibility of amino acids within the sequence.
17. **Blosum3** - Another descriptor based on the BLOSUM62 matrix.
18. **t1** - Part of the **tScales** indices, representing physicochemical properties of amino acids.
19. **Blosum2** - A BLOSUM-based index capturing evolutionary conservation and similarity in amino acid sequences.
20. **t2** - Another **tScales** descriptor.
21. **at_charge** - Theoretical net charge of the sequence at a given pH, based on the Henderson-Hasselbalch equation.
22. **PorcBasic** - Percentage of basic amino acids (H, K, R) in the sequence.
23. **PorcSmall** - Percentage of small amino acids in the sequence.
24. **z5** - Z- scale descriptor related to electronegativity and molecular hardness.
25. **pKC** - pK-C descriptor, likely related to the pKa of cysteine or other ionizable side chains.
26. **AlphaAndTurnPropensities** - Measures the tendency of amino acids to form alpha-helices and turns.
27. **HelixBendPreference** - Represents the propensity of amino acids to bend within a helical structure.
28. **st3** - Another steric property descriptor from **stScales**.
29. **PorcTiny** - Percentage of tiny amino acids (A, C, G, S).
30. **PorcCharged** - Percentage of charged amino acids (both positively and negatively charged).

**Annex 7.** **Sequence counts after CD-HIT filtering**. Increasing the stringency of redundancy filtering (i.e., lowering the identity threshold) reduced the number of retained sequences, particularly in the epitope dataset. This process helps minimize overfitting by increasing sequence diversity, albeit at the cost of reducing training size.

| **CD-HIT Threshold** | **Epitope Sequences** | **Non-Epitope Sequences** |
| --- | --- | --- |
| 95% | 169 | 587 |
| 90% | 157 | 580 |
| 85% | 152 | 577 |
| 80% | 148 | 462 |

**Annex 8:** Metric Tables by CD-HIT Threshold – 95%.

| ALL | Accuracy | Recall | Precision | F1 Score | MCC |
| --- | --- | --- | --- | --- | --- |
| RF | 0.76 ± 0.05 | 0.78 ± 0.06 | 0.68 ± 0.07 | 0.73 ± 0.05 | 0.53 ± 0.10 |
| NB | 0.68 ± 0.05 | 0.73 ± 0.04 | 0.59 ± 0.06 | 0.65 ± 0.04 | 0.38 ± 0.08 |
| DT | 0.65 ± 0.05 | 0.56 ± 0.17 | 0.57 ± 0.08 | 0.55 ± 0.08 | 0.28 ± 0.10 |
| XG | 0.64 ± 0.06 | 0.66 ± 0.14 | 0.55 ± 0.07 | 0.59 ± 0.07 | 0.30 ± 0.11 |
| SVM | 0.58 ± 0.06 | 0.78 ± 0.06 | 0.49 ± 0.05 | 0.60 ± 0.04 | 0.23 ± 0.10 |
|  |  |  |  |  |  |
|  |  |  |  |  |  |
| ANOVA | Accuracy | Recall | Precision | F1 Score | MCC |
| RF | 0.75 ± 0.05 | 0.79 ± 0.05 | 0.66 ± 0.06 | 0.72 ± 0.05 | 0.51 ± 0.09 |
| NB | 0.67 ± 0.05 | 0.79 ± 0.04 | 0.57 ± 0.05 | 0.66 ± 0.04 | 0.38 ± 0.08 |
| DT | 0.62 ± 0.06 | 0.56 ± 0.18 | 0.54 ± 0.08 | 0.53 ± 0.08 | 0.24 ± 0.10 |
| XGB | 0.63 ± 0.05 | 0.71 ± 0.11 | 0.53 ± 0.05 | 0.60 ± 0.06 | 0.29 ± 0.10 |
| SVC | 0.48 ± 0.07 | 0.83 ± 0.14 | 0.42 ± 0.04 | 0.56 ± 0.05 | 0.09 ± 0.13 |
|  |  |  |  |  |  |
| MI | Accuracy | Recall | Precision | F1 Score | MCC |
| RF | 0.76 ± 0.04 | 0.76 ± 0.05 | 0.68 ± 0.06 | 0.72 ± 0.04 | 0.52 ± 0.08 |
| NB | 0.64 ± 0.06 | 0.84 ± 0.06 | 0.53 ± 0.05 | 0.65 ± 0.04 | 0.36 ± 0.09 |
| DT | 0.64 ± 0.06 | 0.54 ± 0.12 | 0.56 ± 0.08 | 0.54 ± 0.07 | 0.26 ± 0.10 |
| XGB | 0.64 ± 0.06 | 0.68 ± 0.11 | 0.55 ± 0.07 | 0.60 ± 0.06 | 0.29 ± 0.11 |
| SVC | 0.46 ± 0.07 | 0.74 ± 0.27 | 0.39 ± 0.07 | 0.50 ± 0.13 | 0.01 ± 0.15 |
|  |  |  |  |  |  |
| MIX | Accuracy | Recall | Precision | F1 Score | MCC |
| RF | 0.76 ± 0.05 | 0.79 ± 0.06 | 0.67 ± 0.06 | 0.72 ± 0.05 | 0.52 ± 0.09 |
| NB | 0.70 ± 0.06 | 0.81 ± 0.04 | 0.60 ± 0.06 | 0.69 ± 0.05 | 0.44 ± 0.09 |
| DT | 0.64 ± 0.06 | 0.55 ± 0.16 | 0.57 ± 0.09 | 0.54 ± 0.07 | 0.27 ± 0.10 |
| XGB | 0.63 ± 0.06 | 0.69 ± 0.12 | 0.54 ± 0.07 | 0.59 ± 0.05 | 0.28 ± 0.10 |
| SVC | 0.54 ± 0.09 | 0.81 ± 0.11 | 0.46 ± 0.06 | 0.58 ± 0.05 | 0.18 ± 0.15 |

**Annex 9:** Metric Tables by CD-HIT Threshold – 90%.

| ALL | Accuracy | Recall | Precision | F1 Score | MCC |
| --- | --- | --- | --- | --- | --- |
| RF | 0.75 ± 0.04 | 0.74 ± 0.05 | 0.73 ± 0.06 | 0.73 ± 0.04 | 0.51 ± 0.09 |
| NB | 0.64 ± 0.05 | 0.64 ± 0.04 | 0.61 ± 0.06 | 0.62 ± 0.04 | 0.28 ± 0.10 |
| DT | 0.65 ± 0.06 | 0.59 ± 0.16 | 0.65 ± 0.10 | 0.60 ± 0.07 | 0.31 ± 0.12 |
| XG | 0.64 ± 0.05 | 0.65 ± 0.11 | 0.62 ± 0.07 | 0.63 ± 0.06 | 0.30 ± 0.10 |
| SVM | 0.58 ± 0.05 | 0.61 ± 0.05 | 0.55 ± 0.06 | 0.57 ± 0.04 | 0.17 ± 0.10 |
|  |  |  |  |  |  |
|  |  |  |  |  |  |
| ANOVA | Accuracy | Recall | Precision | F1 Score | MCC |
| RF | 0.72 ± 0.05 | 0.67 ± 0.06 | 0.71 ± 0.07 | 0.69 ± 0.05 | 0.44 ± 0.10 |
| NB | 0.62 ± 0.04 | 0.74 ± 0.05 | 0.57 ± 0.04 | 0.64 ± 0.03 | 0.26 ± 0.08 |
| DT | 0.63 ± 0.05 | 0.56 ± 0.15 | 0.62 ± 0.08 | 0.57 ± 0.07 | 0.27 ± 0.10 |
| XGB | 0.64 ± 0.06 | 0.68 ± 0.09 | 0.60 ± 0.06 | 0.64 ± 0.06 | 0.29 ± 0.11 |
| SVC | 0.54 ± 0.06 | 0.61 ± 0.08 | 0.51 ± 0.05 | 0.55 ± 0.04 | 0.09 ± 0.11 |
|  |  |  |  |  |  |
| MI | Accuracy | Recall | Precision | F1 Score | MCC |
| RF | 0.75 ± 0.04 | 0.69 ± 0.05 | 0.74 ± 0.06 | 0.71 ± 0.04 | 0.49 ± 0.08 |
| NB | 0.63 ± 0.05 | 0.83 ± 0.04 | 0.57 ± 0.04 | 0.68 ± 0.04 | 0.31 ± 0.09 |
| DT | 0.66 ± 0.05 | 0.52 ± 0.08 | 0.68 ± 0.09 | 0.58 ± 0.06 | 0.32 ± 0.10 |
| XGB | 0.62 ± 0.06 | 0.61 ± 0.10 | 0.59 ± 0.07 | 0.59 ± 0.06 | 0.24 ± 0.11 |
| SVC | 0.57 ± 0.05 | 0.65 ± 0.07 | 0.53 ± 0.05 | 0.58 ± 0.05 | 0.16 ± 0.10 |
|  |  |  |  |  |  |
| MIX | Accuracy | Recall | Precision | F1 Score | MCC |
| RF | 0.73 ± 0.05 | 0.68 ± 0.06 | 0.72 ± 0.07 | 0.70 ± 0.05 | 0.45 ± 0.10 |
| NB | 0.63 ± 0.05 | 0.71 ± 0.04 | 0.59 ± 0.05 | 0.64 ± 0.04 | 0.28 ± 0.09 |
| DT | 0.62 ± 0.05 | 0.56 ± 0.15 | 0.61 ± 0.07 | 0.57 ± 0.07 | 0.25 ± 0.09 |
| XGB | 0.64 ± 0.06 | 0.67 ± 0.10 | 0.60 ± 0.06 | 0.63 ± 0.06 | 0.28 ± 0.11 |
| SVC | 0.55 ± 0.04 | 0.60 ± 0.06 | 0.51 ± 0.04 | 0.55 ± 0.03 | 0.11 ± 0.08 |

**Annex 10:** Metric Tables by CD-HIT Threshold – 85%.

| ALL | Accuracy | Recall | Precision | F1 Score | MCC |
| --- | --- | --- | --- | --- | --- |
| RF | 0.78 ± 0.05 | 0.79 ± 0.05 | 0.81 ± 0.06 | 0.80 ± 0.05 | 0.57 ± 0.10 |
| NB | 0.74 ± 0.04 | 0.80 ± 0.04 | 0.74 ± 0.05 | 0.77 ± 0.03 | 0.48 ± 0.08 |
| DT | 0.67 ± 0.06 | 0.64 ± 0.12 | 0.73 ± 0.08 | 0.67 ± 0.07 | 0.36 ± 0.12 |
| XG | 0.68 ± 0.06 | 0.73 ± 0.10 | 0.69 ± 0.06 | 0.71 ± 0.06 | 0.35 ± 0.11 |
| SVM | 0.62 ± 0.08 | 0.68 ± 0.13 | 0.64 ± 0.09 | 0.65 ± 0.11 | 0.24 ± 0.16 |
|  |  |  |  |  |  |
|  |  |  |  |  |  |
| ANOVA | Accuracy | Recall | Precision | F1 Score | MCC |
| RF | 0.74 ± 0.04 | 0.67 ± 0.06 | 0.75 ± 0.07 | 0.71 ± 0.05 | 0.47 ± 0.09 |
| NB | 0.71 ± 0.05 | 0.82 ± 0.03 | 0.66 ± 0.06 | 0.73 ± 0.04 | 0.44 ± 0.09 |
| DT | 0.66 ± 0.05 | 0.53 ± 0.09 | 0.70 ± 0.08 | 0.59 ± 0.06 | 0.33 ± 0.10 |
| XGB | 0.64 ± 0.06 | 0.65 ± 0.11 | 0.63 ± 0.08 | 0.63 ± 0.07 | 0.29 ± 0.13 |
| SVC | 0.58 ± 0.05 | 0.70 ± 0.07 | 0.55 ± 0.05 | 0.61 ± 0.04 | 0.17 ± 0.10 |
|  |  |  |  |  |  |
| MI | Accuracy | Recall | Precision | F1 Score | MCC |
| RF | 0.76 ± 0.05 | 0.74 ± 0.06 | 0.75 ± 0.08 | 0.74 ± 0.05 | 0.52 ± 0.11 |
| NB | 0.75 ± 0.04 | 0.77 ± 0.03 | 0.72 ± 0.05 | 0.74 ± 0.03 | 0.50 ± 0.07 |
| DT | 0.65 ± 0.06 | 0.60 ± 0.13 | 0.67 ± 0.09 | 0.62 ± 0.07 | 0.32 ± 0.11 |
| XGB | 0.65 ± 0.06 | 0.67 ± 0.12 | 0.63 ± 0.07 | 0.64 ± 0.07 | 0.30 ± 0.12 |
| SVC | 0.57 ± 0.06 | 0.68 ± 0.08 | 0.54 ± 0.05 | 0.60 ± 0.05 | 0.16 ± 0.12 |
|  |  |  |  |  |  |
| MIX | Accuracy | Recall | Precision | F1 Score | MCC |
| RF | 0.76 ± 0.04 | 0.72 ± 0.05 | 0.77 ± 0.06 | 0.74 ± 0.04 | 0.53 ± 0.08 |
| NB | 0.73 ± 0.04 | 0.74 ± 0.03 | 0.71 ± 0.06 | 0.72 ± 0.03 | 0.46 ± 0.08 |
| DT | 0.65 ± 0.06 | 0.65 ± 0.13 | 0.65 ± 0.09 | 0.64 ± 0.06 | 0.32 ± 0.12 |
| XGB | 0.66 ± 0.05 | 0.69 ± 0.11 | 0.64 ± 0.07 | 0.66 ± 0.05 | 0.33 ± 0.10 |
| SVC | 0.58 ± 0.05 | 0.66 ± 0.07 | 0.55 ± 0.05 | 0.60 ± 0.05 | 0.17 ± 0.10 |

**Annex 11:** Metric Tables by CD-HIT Threshold – 80%.

| ALL | Accuracy | Recall | Precision | F1 Score | MCC |
| --- | --- | --- | --- | --- | --- |
| RF | 0.72 ± 0.05 | 0.68 ± 0.05 | 0.77 ± 0.06 | 0.72 ± 0.04 | 0.46 ± 0.09 |
| NB | 0.66 ± 0.05 | 0.69 ± 0.03 | 0.67 ± 0.05 | 0.68 ± 0.03 | 0.33 ± 0.09 |
| DT | 0.65 ± 0.06 | 0.61 ± 0.10 | 0.70 ± 0.09 | 0.64 ± 0.06 | 0.31 ± 0.12 |
| XG | 0.63 ± 0.05 | 0.68 ± 0.08 | 0.64 ± 0.06 | 0.65 ± 0.05 | 0.26 ± 0.11 |
| SVM | 0.60 ± 0.05 | 0.61 ± 0.11 | 0.61 ± 0.07 | 0.60 ± 0.10 | 0.20 ± 0.10 |
|  |  |  |  |  |  |
|  |  |  |  |  |  |
| ANOVA | Accuracy | Recall | Precision | F1 Score | MCC |
| RF | 0.71 ± 0.05 | 0.68 ± 0.06 | 0.75 ± 0.06 | 0.71 ± 0.05 | 0.43 ± 0.09 |
| NB | 0.60 ± 0.05 | 0.65 ± 0.04 | 0.62 ± 0.06 | 0.63 ± 0.04 | 0.21 ± 0.11 |
| DT | 0.64 ± 0.06 | 0.63 ± 0.13 | 0.68 ± 0.10 | 0.64 ± 0.06 | 0.30 ± 0.13 |
| XGB | 0.63 ± 0.06 | 0.68 ± 0.09 | 0.64 ± 0.07 | 0.66 ± 0.05 | 0.27 ± 0.12 |
| SVC | 0.57 ± 0.07 | 0.58 ± 0.22 | 0.55 ± 0.15 | 0.55 ± 0.19 | 0.14 ± 0.14 |
|  |  |  |  |  |  |
| MI | Accuracy | Recall | Precision | F1 Score | MCC |
| RF | 0.73 ± 0.05 | 0.69 ± 0.06 | 0.76 ± 0.07 | 0.72 ± 0.05 | 0.46 ± 0.10 |
| NB | 0.68 ± 0.05 | 0.67 ± 0.05 | 0.69 ± 0.06 | 0.68 ± 0.04 | 0.35 ± 0.09 |
| DT | 0.63 ± 0.06 | 0.57 ± 0.13 | 0.68 ± 0.07 | 0.61 ± 0.08 | 0.28 ± 0.12 |
| XGB | 0.63 ± 0.06 | 0.66 ± 0.10 | 0.64 ± 0.06 | 0.65 ± 0.07 | 0.27 ± 0.12 |
| SVC | 0.63 ± 0.06 | 0.69 ± 0.11 | 0.63 ± 0.05 | 0.65 ± 0.08 | 0.26 ± 0.11 |
|  |  |  |  |  |  |
| MIX | Accuracy | Recall | Precision | F1 Score | MCC |
| RF | 0.72 ± 0.05 | 0.69 ± 0.06 | 0.75 ± 0.06 | 0.72 ± 0.05 | 0.44 ± 0.10 |
| NB | 0.66 ± 0.05 | 0.72 ± 0.02 | 0.66 ± 0.06 | 0.69 ± 0.03 | 0.32 ± 0.10 |
| DT | 0.65 ± 0.06 | 0.63 ± 0.13 | 0.68 ± 0.09 | 0.64 ± 0.08 | 0.30 ± 0.13 |
| XGB | 0.64 ± 0.06 | 0.69 ± 0.10 | 0.64 ± 0.06 | 0.66 ± 0.06 | 0.28 ± 0.11 |
| SVC | 0.61 ± 0.05 | 0.64 ± 0.11 | 0.61 ± 0.10 | 0.62 ± 0.10 | 0.22 ± 0.10 |

**Annex 12:** Performance of BepiPred-3.0 on our dataset using the official classification threshold (0.1512).

| **Metric** | **Value** |
| --- | --- |
| TPR (Sensitivity) | 0.974 |
| FPR (False Positive Rate) | 0.926 |
| TNR (Specificity) | 0.074 |
| PPV (Precision) | 0.627 |
| Negative Predictive Value (NPV) | 0.200 |
| Balanced Accuracy | 0.524 |
| Threshold used | 0.1512 |

BepiPred-3.0 was evaluated using its recommended default threshold of 0.1512, as indicated in the original publication. At this threshold, the method classified 97.4% of positive residues and 92.6% of negative residues as epitopes, resulting in very limited separation between the two classes. Despite a high true-positive rate, the extremely elevated false-positive rate (92.6%) led to a low specificity and a balanced accuracy close to random performance. These results demonstrate that BepiPred-3.0 exhibits minimal discriminative power on our dataset when used as a binary classifier, supporting the need for alternative models capable of providing better class separation.

**Annex 13:** Performance of iBCE-EL (PIP-EL mode) on our benchmark dataset.

| **Metric** | **Value** |
| --- | --- |
| True Positives (TP) | 68 |
| False Negatives (FN) | 84 |
| True Negatives (TN) | 107 |
| False Positives (FP) | 45 |
| Sensitivity (TPR) | 0.447 |
| Specificity (TNR) | 0.704 |
| Accuracy | 0.576 |
| Precision (PPV) | 0.602 |
| Negative Predictive Value (NPV) | 0.560 |
| Matthews Correlation Coefficient (MCC) | 0.157 |

**Annex 14:** Classification performance of EpitopeVec on the balanced positive/negative Influenza epitope dataset.

| **Metric** | **Value** |
| --- | --- |
| TP | 65 |
| FN | 87 |
| FP | 23 |
| TN | 129 |
| Sensitivity (TPR) | 0.428 |
| Specificity (TNR) | 0.849 |
| False Positive Rate (FPR) | 0.151 |
| Accuracy | 0.638 |
| Precision (PPV) | 0.739 |
| Negative Predictive Value (NPV) | 0.597 |
| Balanced Accuracy | 0.638 |
| MCC | 0.304 |

**Annex 15**: Sensitivity Analysis. The classification performance is influenced by peptide length during the generation of artificial negative samples. A Random Forest model was employed for the analysis.

| **Peptide length (aa)** | **Accuracy** | **Balanced Accuracy** | **MCC** |
| --- | --- | --- | --- |
| 12 | 0.808 ± 0.044 | 0.808 ± 0.043 | 0.618 ± 0.087 |
| 14 | 0.715 ± 0.044 | 0.713 ± 0.043 | 0.430 ± 0.088 |
| 16 | 0.648 ± 0.054 | 0.645 ± 0.054 | 0.295 ± 0.110 |

**Annex 16:** hyperparameters

All machine learning models were implemented in Python using the **scikit-learn** library, except for XGBoost which was implemented using the **xgboost** package. Hyperparameters were selected following commonly used configurations reported in the literature and optimized through cross-validation during model training. The main parameters explored for each algorithm are summarized below.

**Naive Bayes (NB)**

The Gaussian Naive Bayes classifier was implemented using the **GaussianNB** class from scikit-learn. The parameter var_smoothing was evaluated to stabilize variance estimates and improve numerical stability.

Evaluated parameters:

- var_smoothing ∈ {1e-9, 1e-8, 1e-7}

Final configuration:

- var_smoothing = 1e-9

**Random Forest (RF)**

The Random Forest classifier was implemented using the **RandomForestClassifier** from scikit-learn. The number of trees and node splitting parameters were explored to balance model complexity and overfitting prevention.

Evaluated parameters:

- n_estimators ∈ {100, 300, 500}
- min_samples_split ∈ {2, 5, 10}
- min_samples_leaf ∈ {1, 2, 4}

Final configuration:

- n_estimators = 100
- min_samples_split = 2
- min_samples_leaf = 1
- random_state = 123

**XGBoost (XG)**

Gradient boosting models were implemented using the **XGBoost** library with the objective function set to binary classification (binary:logistic).

Evaluated parameters:

- n_estimators ∈ {2, 10, 50}
- max_depth ∈ {2, 3, 5}
- learning_rate ∈ {0.1, 0.5, 1}

Final configuration:

- n_estimators = 2
- max_depth = 2
- learning_rate = 1

**Decision Tree (DT)**

Decision trees were implemented using **DecisionTreeClassifier** from scikit-learn. A conservative split strategy was applied to reduce the risk of overfitting given the relatively small dataset size.

Evaluated parameters:

- min_samples_split ∈ {50, 100, 200}
- max_depth ∈ {None, 10, 20}

Final configuration:

- min_samples_split = 100
- random_state = 123

**Support Vector Machine (SVM)**

The Support Vector Machine classifier was implemented using an RBF kernel. Hyperparameter optimization explored different values for the regularization parameter (C) and kernel coefficient (γ). The final model used probability=True to enable probabilistic output required for downstream evaluation metrics and random_state=123 to ensure reproducibility.

Evaluated parameters:

- C ∈ {0.1, 1, 10, 100}
- gamma ∈ {scale, 0.001, 0.01, 0.1}
- kernel = RBF

The optimal configuration for each model was selected based on cross-validation performance during training.
